# Structural Engineering Modulation of Metal Sulfide Photocatalysts from Charge Regulation Mechanisms to Practical Applications

**DOI:** 10.3390/molecules31203666

**Published:** 2026-10-13

**Authors:** Guangteng Bai, Xi Luo, Zhao Mo, Xu Liu, Zaiyong Jiang, Xianglin Zhu

**Affiliations:** 1 Institute for Energy Research, Jiangsu University, Zhenjiang 212013, China; 2 School of Chemistry & Chemical Engineering and Environmental Engineering, Weifang University, Weifang 261061, China

**Keywords:** metal sulfides, structural engineering, charge-carrier dynamics, photocatalysis, energy and environmental applications

## Abstract

Metal sulfide semiconductors have attracted considerable attention in solar-driven energy conversion and environmental remediation because of their strong visible-light response, tunable electronic structures, and abundant surface-active sites. Nevertheless, further improvements in their photocatalytic performance remain constrained by rapid charge-carrier recombination, photocorrosion, insufficient reaction selectivity, and limited operational stability under practical conditions. Existing reviews have largely focused on specific sulfide materials, individual photocatalytic reactions, or a single structural-regulation strategy, whereas the intrinsic relationships among material composition, structural engineering, charge-carrier dynamics, and application requirements have not been systematically examined. Following the conceptual framework of “material composition–structural regulation–charge-carrier dynamics–reaction pathways–engineering applications,” this review provides a systematic overview of the design principles and application advances of metal sulfide photocatalysts. First, the crystal structures, band characteristics, surface-active sites, and photocorrosion behavior of different classes of metal sulfides are analyzed to clarify how their intrinsic properties govern catalytic processes. The effects of morphology engineering, defect regulation, compositional optimization, interface construction, and cocatalyst modification on light absorption, charge transport, and surface reaction kinetics are then discussed, with particular attention to how these strategies balance activity enhancement and stability retention. The distinctive features of metal sulfides in photocatalytic hydrogen evolution, biomass photoreforming, CO_2_ reduction, H_2_O_2_ synthesis, pollutant degradation, and photoelectrochemical sensing are further summarized, revealing the distinct requirements of different reaction systems for electronic structures and active sites. Finally, current challenges and future research directions are discussed in terms of mechanistic reliability, material stability, adaptability to realistic systems, and sustainable development. This review aims to establish an application-oriented framework for the rational design of metal sulfide photocatalysts and to provide a theoretical basis for developing efficient, stable, and sustainable photocatalytic systems.

## 1. Introduction

With the continued growth in energy demand and the increasing severity of environmental pollution, green energy conversion and pollution remediation have attracted extensive attention [1,2,3,4,5,6,7,8,9,10,11,12,13,14,15]. Photocatalysis enables photogenerated charge carriers in semiconductor materials to participate in redox reactions, thereby offering considerable potential for solar hydrogen production, CO_2_ valorization, N_2_ fixation and pollutant degradation [16,17,18,19,20,21,22,23]. Nevertheless, conventional semiconductor materials remain constrained by limited light-harvesting ranges, rapid charge-carrier recombination, and sluggish surface reaction kinetics, leaving substantial scope for improving their practical solar-energy conversion efficiency [24,25,26].

Metal sulfides have been widely investigated for energy conversion and environmental remediation because of their relatively narrow bandgaps, strong visible-light response, and tunable electronic structures [27,28,29,30,31]. According to their elemental composition, these materials can be broadly classified as binary, ternary, or multicomponent sulfide systems. Pronounced differences in band structure, spectral response, and stability exist among these categories; their intrinsic properties and the characteristics of representative materials are discussed in subsequent sections [32,33,34]. To overcome the performance limitations of single-component sulfides, researchers have developed multinary sulfides and solid-solution materials in which adjustment of the elemental composition allows the electronic structure and band-edge positions to be optimized. For example, the bandgap of ZnCdS solid solutions can be continuously tuned by varying the Zn/Cd ratio. This compositional flexibility improves charge-carrier transport while preserving a strong reduction capability, making ZnCdS an important material platform for the hydrogen evolution reaction (HER) [24,35]. Multinary sulfides such as ZnIn_2_S_4_, In_2_S_2_, and CuInS_2_ also exhibit considerable versatility in photocatalytic energy conversion and environmental remediation owing to their layered structures, synergistic interactions among multiple metal species, and diverse surface chemistry [36,37]. Composition regulation alone, however, cannot fully suppress photogenerated charge-carrier recombination or resolve the limitations imposed by sluggish surface reaction kinetics [38].

In recent years, research emphasis has gradually shifted from compositional optimization toward defect engineering, interface engineering, and multiscale structural regulation [38,39]. Defects such as sulfur and metal vacancies can redistribute local electron density and modulate surface adsorption behavior [40,41,42,43,44]. Heterostructure construction, by contrast, facilitates the directional migration of photogenerated charge carriers through interfacial band alignment and built-in electric fields [45,46,47]. Further advances involving S-scheme and Z-scheme heterojunctions, single-atom cocatalysts, and synergistic defect–interface regulation have opened new avenues for enhancing the photocatalytic performance of metal sulfides [48,49,50]. Despite rapid progress in metal sulfide photocatalysts, most existing reviews remain centered on a single material system, reaction type, or structural-regulation strategy [50,51,52,53]. Some studies, for instance, focus primarily on CdS-based systems for photocatalytic hydrogen evolution, whereas others emphasize the development of layered sulfides such as ZnIn_2_S_4_ [54,55]. A systematic account of the relationships among material composition, structural regulation, charge-carrier dynamics, and practical application requirements is still lacking [27]. This limitation is particularly relevant when both energy conversion and environmental remediation are considered, as different reactions impose distinct requirements on the electronic structure, surface-active sites, and charge-transport pathways of a catalyst [24]. HER generally requires a sufficiently negative conduction-band position and efficient electron transport. In contrast, CO_2_ reduction, H_2_O_2_ production, and the oxidative degradation of pollutants depend more strongly on the adsorption of reaction intermediates, the regulation of surface-active sites, and the optimization of redox pathways [56,57,58]. Classification based solely on material type or application area therefore cannot fully reveal the intrinsic relationships among structural design, charge-carrier behavior, and reaction selectivity [27].

Moreover, a substantial proportion of current research is still conducted in idealized experimental systems, with insufficient attention given to engineering considerations such as complex aqueous matrices, catalyst immobilization, long-term operational stability, and life-cycle assessment [59]. Photocatalysis has recently begun to converge with research on agricultural-waste-derived carbon materials, biomass valorization, and the treatment of complex agricultural water matrices. These emerging directions offer new opportunities for the immobilization and practical deployment of metal sulfide catalysts [60,61].

**Figure 1:**
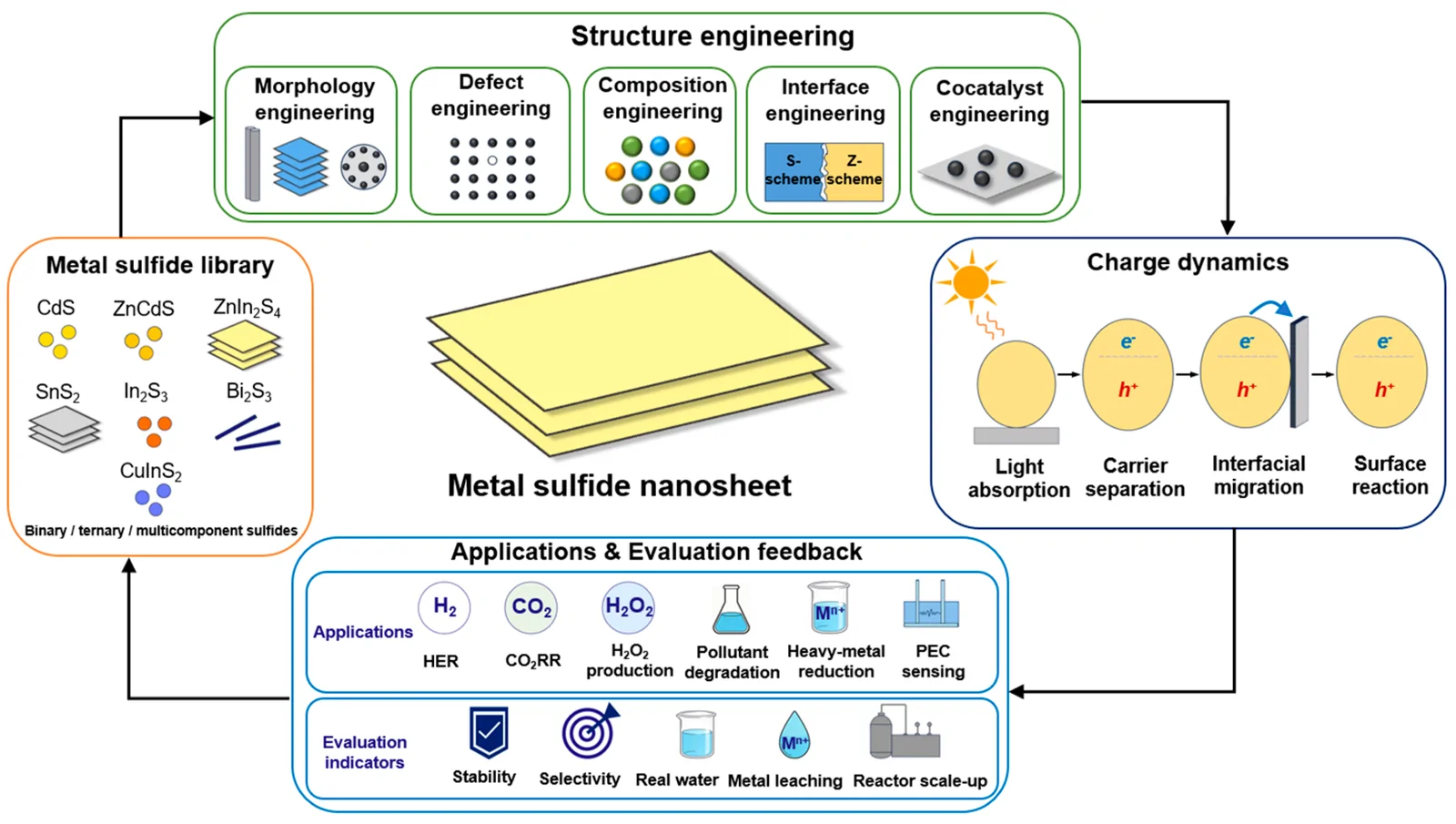
Overall framework linking material composition, structural regulation, charge dynamics, and energy/environmental applications of metal sulfide photocatalysts.

In view of these issues, this review systematically examines the development of metal sulfide photocatalysts along the conceptual framework of “intrinsic material properties–structural regulation strategies–charge-carrier dynamics–photocatalytic applications.” As illustrated in Figure 1, structural regulation forms the central theme of the review, followed by a sequential discussion of the intrinsic properties of metal sulfides, their photocatalytic applications across multiple scenarios, and the associated engineering challenges. Particular attention is devoted to the potential value of agricultural-waste-derived supports and metal sulfide photocatalysts for treating complex agricultural water matrices under practical conditions. By integrating these topics, this review seeks to facilitate the transition of metal sulfide photocatalysis from laboratory-scale investigation to practical application [62].

Unlike reviews organized around a single sulfide, reaction, or modification method, the present review connects intrinsic band and surface properties with structural regulation, charge-carrier behavior, reaction selectivity, and engineering constraints. This organization allows readers to compare why a given strategy works for one reaction but may be ineffective for another, and to identify where reported activity gains are offset by photocorrosion, donor dependence, or poor performance in real matrices. The review also emphasizes evidence standards, including operando verification and comparable reporting, to help distinguish demonstrated mechanisms from proposed ones.

## 2. Fundamental Properties, Charge-Carrier Dynamics, and Mechanistic Understanding

The photocatalytic performance of metal sulfides depends not only on their composition and structural characteristics but also on the combined effects of band structure, surface-active sites, charge-carrier transport, and interfacial reaction processes. An understanding of their intrinsic electronic structures and photocatalytic processes is therefore essential for subsequent structural regulation and application-oriented design [28]. Following the conceptual sequence of “composition and structure–electronic properties–charge-carrier behavior–surface reactions–stability limitations,” this section summarizes the principal factors governing photocatalytic processes in metal sulfides [27].

### 2.1. Crystal Structures and Electronic Properties of Metal Sulfides

Metal sulfides encompass a wide range of compositions and crystal structures. Different combinations of metal elements confer tunable electronic structures and optical properties, which are critical determinants of photocatalytic performance [28,63]. Based on their elemental composition, metal sulfides can be broadly categorized as binary sulfides, ternary or multinary sulfides, and multicomponent sulfide systems. These categories exhibit pronounced differences in bandgap, band-edge positions, and surface electronic states. Binary sulfides were among the earliest material systems investigated in metal sulfide photocatalysis, with CdS and ZnS being the most representative examples [55].

CdS has a bandgap of approximately 2.4 eV and a relatively negative conduction-band position, thereby satisfying the thermodynamic requirement for proton reduction during photocatalytic hydrogen evolution. It has consequently long served as an important model material for visible-light-driven hydrogen production [55,64]. However, its strong photogenerated-electron reduction capability is accompanied by pronounced susceptibility to photocorrosion. Under prolonged irradiation, oxidation of lattice sulfur and the release of Cd^2+^ ions can readily occur, resulting in a progressive loss of catalytic activity [65,66]. In comparison, ZnS exhibits higher chemical stability, but its wide bandgap limits visible-light absorption. Elemental doping, defect regulation, or the construction of composite architectures is therefore generally required to improve the catalytic activity [67,68,69,70]. The development of binary sulfides laid an important foundation for research into metal sulfide photocatalysis, while simultaneously revealing the difficulty of balancing catalytic activity, spectral response, and stability [55].

To further optimize the electronic structure, increasing attention has been directed toward multinary sulfides and solid-solution materials [71,72]. The bandgap of ZnCdS can be continuously tuned by varying the Zn/Cd ratio, enabling improved charge-carrier transport while retaining a strong reduction capability. ZnCdS has thus become an important material system for studies of the hydrogen evolution reaction (HER) [24,35]. Moreover, multinary sulfides such as ZnIn_2_S_4_, In_2_S_3_, SnS_2_, and CuInS_2_ exhibit considerable versatility in photocatalytic energy conversion and environmental remediation because of their layered structures, synergistic interactions among multiple metal species, and diverse surface chemical environments.

As summarized in Table 1, differences in band structure and surface properties make individual metal sulfides suitable for distinct types of photocatalytic reactions. CdS and ZnCdS, for example, possess relatively negative conduction-band positions and are therefore better suited to electron-driven reduction reactions. By contrast, materials such as ZnIn_2_S_4_ and SnS_2_ show considerable potential for CO_2_ reduction, H_2_O_2_ production, and pollutant degradation owing to their stable structures and tunable surface properties [56,57,58].

Table 1 should be read as a comparison of representative ranges rather than a strict ranking: band gaps and band edges vary with crystal phase, composition, morphology, defect concentration, and measurement method. In addition to CdS and ZnS, binary systems such as MoS_2_, WS_2_, and SnS_2_ and ternary systems such as ZnIn_2_S_4_ and CuInS_2_ span visible-light-relevant optical gaps. Reported values are therefore given as approximate literature ranges, and band-edge values should be compared only when measured or calculated under compatible conditions.

### 2.2. Surface-Active Sites and Reaction-Related Electronic Structures

Photocatalytic reactions occur predominantly at material surfaces; consequently, the nature and electronic states of surface-active sites, together with their capacity to adsorb reactants, directly influence the catalytic process [73,74,75]. In metal sulfides, metal sites, sulfur sites, and defect-induced coordinatively unsaturated sites may all participate in surface reactions. These sites affect catalytic performance by regulating reactant adsorption and the stability of reaction intermediates [41].

During the hydrogen evolution reaction (HER), metal active sites generally participate in proton adsorption and the formation of adsorbed hydrogen intermediates (H*), while their electronic structures determine the Gibbs free energy of hydrogen adsorption (ΔG_H*_). An effective catalyst must maintain an appropriate balance between H* adsorption and desorption because either excessively strong or overly weak binding can impair reaction kinetics [48,76]. In CO_2_ reduction and H_2_O_2_ production, by contrast, reactant activation and the stability of key redox intermediates are major determinants of the operative reaction pathways [57,77,78,79].

In addition to intrinsic active sites, lattice defects play an important role in regulating the surface electronic environment. Defects such as sulfur vacancies can induce changes in localized electronic states and alter the coordination environments of neighboring atoms, thereby modifying reactant adsorption. Their effects are not uniformly beneficial, however. An appropriate defect concentration can generate effective active centers, whereas excessive defect formation may compromise structural stability or introduce additional charge-carrier recombination centers [41,80].

Surface chemistry further determines whether these sites are accessible and selective. Adsorbed water, hydroxyl groups, pH-dependent protonation, and specifically adsorbed anions can change the coordination and charge of exposed metal centers. For CO_2_ reduction, surface hydroxylation and local proton availability affect the stabilization of *COOH and *OCHO; for oxygen reduction, the binding geometry and proton-coupled electron transfer steps determine whether O–O cleavage is avoided. Consequently, adsorption energies calculated for ideal, dry surfaces may not describe the working interface. Explicit solvent, surface coverage, and competing adsorbates should be considered where they can alter the predicted rate-limiting step.

### 2.3. Charge-Carrier Generation, Transport, and Recombination Dynamics

The generation, transport, and recombination of photogenerated charge carriers are key processes governing the photocatalytic efficiency of metal sulfides [81,82,83,84,85]. Upon photon absorption, a semiconductor generates electron–hole pairs that subsequently undergo a series of processes, including bulk transport, interfacial transfer, and surface reactions. Charge loss at any of these stages can diminish the overall photocatalytic efficiency [81]. A detailed understanding of charge-carrier dynamics is therefore fundamental to the rational design of efficient metal sulfide photocatalysts [86].

Under practical photocatalytic conditions, photogenerated electrons and holes often undergo rapid recombination, causing a substantial fraction of the absorbed energy to dissipate as heat and thereby limiting solar-energy utilization [81]. The utilization efficiency of photogenerated charge carriers is governed primarily by the extent of charge loss during their generation, transport, and participation in surface reactions. Because electron–hole recombination commonly occurs during bulk transport and interfacial transfer, charge-carrier lifetime, transport pathways, and interfacial transfer efficiency are major determinants of photocatalytic performance. Elucidating these dynamic processes is thus essential for establishing the relationship between the structural characteristics and catalytic performance of metal sulfides.

Photogenerated charge-carrier dynamics are commonly investigated using steady-state photoluminescence (PL) [87,88], time-resolved photoluminescence (TRPL) [89,90], electrochemical impedance spectroscopy (EIS), transient photocurrent measurements, and surface photovoltage spectroscopy (SPV) [91,92]. Changes in PL intensity provide information about electron–hole recombination behavior, whereas variations in TRPL decay lifetime are used to characterize charge-carrier lifetimes. TA spectroscopy can further resolve charge-transfer dynamics on ultrafast timescales [93,94,95].

### 2.4. Surface Reaction Pathways and Theoretical Descriptors

Photocatalytic performance is governed not only by the generation and transport efficiencies of photogenerated charge carriers but also by surface reaction pathways and the conversion of key intermediates [81]. Combining theoretical calculations with experimental characterization can reveal changes in electronic structure, reactant adsorption behavior, and reaction-energy barriers during metal sulfide catalysis at the atomic scale [96]. In particular, density functional theory (DFT) calculations have become an important tool for elucidating reaction mechanisms [97,98,99,100,101,102].

For HER, the Gibbs free energy of hydrogen adsorption (ΔG_H*_) is a key descriptor of catalytic activity [48,103]. An ideal HER catalyst should exhibit a ΔG_H*_ value close to zero, which favors proton adsorption without binding the resulting hydrogen intermediate so strongly that its desorption becomes kinetically hindered. Analysis of H* adsorption and variations in reaction-energy barriers can therefore clarify the relationship between the surface electronic structure of a catalyst and its hydrogen-evolution kinetics [41,104].

This descriptor follows the Sabatier principle: hydrogen must bind strongly enough for proton/electron transfer and H* formation, yet weakly enough to permit H_2_ release and regenerate the active site. A ΔG_H*_ value near zero is therefore a useful thermodynamic target, but it is not a complete activity predictor. Solvation, pH, applied or local potential, surface coverage, and the kinetic barriers of the Volmer, Heyrovsky, and Tafel steps can shift the optimum. Comparisons among sulfides should use the same computational reference and surface model and be checked against measured rates and stability.

For CO_2_ reduction, the adsorption of CO_2_ on the catalyst surface, its mode of molecular activation, and the stability of key intermediates collectively govern the reaction pathway and product formation. DFT calculations commonly evaluate the CO_2_ adsorption energy and the formation-energy barriers of key intermediates such as COOH* and CO*, thereby revealing electron-transfer and bond-activation behavior during the reaction [77,79,105].

During H_2_O_2_ production, the oxygen reduction pathway and the adsorption behavior of O_2_-derived intermediates are critical to reaction selectivity. Oxygen adsorption energies and intermediate-formation barriers can therefore be used to evaluate the ability of a catalyst to direct the reaction toward the desired pathway [106,107].

These descriptors are useful only when interpreted together with kinetic and selectivity measurements. For example, a favorable adsorption energy does not by itself demonstrate a fast-overall reaction if proton transfer, product desorption, or charge delivery is limiting. Isotope labeling, radical-scavenging controls, product quantification, and operando detection of adsorbed intermediates can help distinguish a proposed pathway from alternative routes. Surface reconstruction should also be considered because the active surface under illumination may differ from the pristine structure used in a calculation.

Beyond reaction-pathway analysis, theoretical calculations can predict adsorption behavior at different active sites, associated changes in electronic structure, and reaction-energy barriers [108]. These insights provide a theoretical basis for understanding the relationships between structural characteristics and catalytic performance [46,109]. Accordingly, structure design guided by theoretical descriptors can facilitate a transition from empirical optimization toward target-oriented design, thereby supporting the development of efficient metal sulfide photocatalysts [62,97].

### 2.5. Operando Characterization and Mechanistic Verification

Because photocatalytic processes involve dynamic charge transfer, surface adsorption, and structural evolution, conventional ex situ characterization alone cannot fully elucidate catalytic mechanisms under realistic reaction conditions. In situ and operando characterization techniques have therefore become increasingly important for investigating photocatalytic processes [65,97].

In situ X-ray photoelectron spectroscopy (XPS) can monitor changes in elemental oxidation states and electron-transfer behavior under irradiation, making it useful for investigating surface electronic reconstruction and photocorrosion in metal sulfides [66,109]. X-ray absorption spectroscopy (XAS) provides complementary information on local coordination environments and electronic structures, thereby facilitating an understanding of the dynamic evolution of metal sites during catalysis [46]. In addition, techniques such as in situ diffuse reflectance infrared Fourier transform spectroscopy (DRIFTS), Raman spectroscopy, and electron paramagnetic resonance (EPR) spectroscopy can detect the formation of adsorbed intermediates and reactive radical species during a reaction. These observations provide direct evidence for elucidating the pathways of CO_2_ reduction, H_2_O_2_ production, and pollutant degradation [77,106,110].

**Figure 2:**
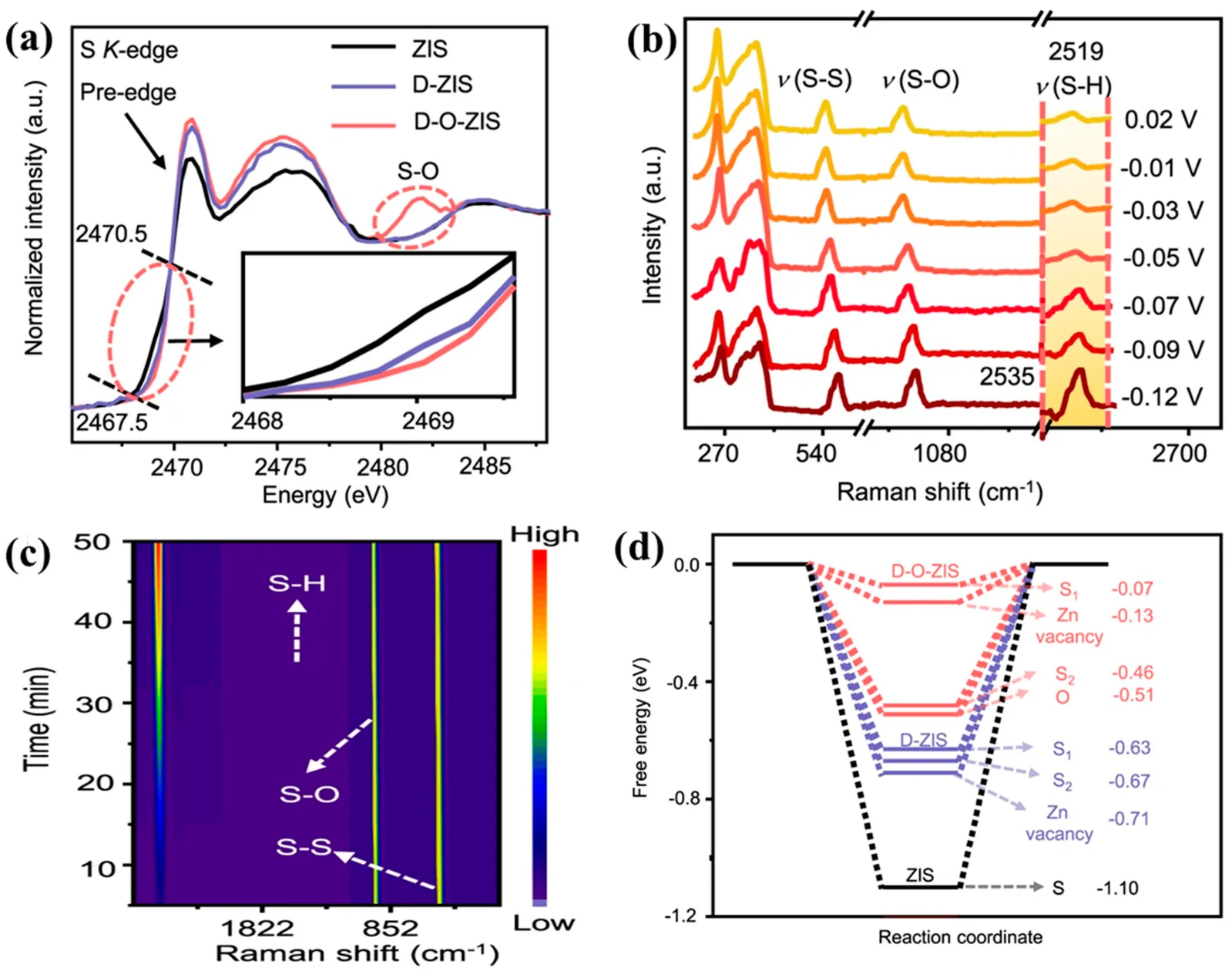
(**a**) Normalized sulfur K-edge XANES spectra of ZIS, D-ZIS and D-O-ZIS samples, (**b**) In situ Raman spectra recorded during the photocatalytic hydrogen evolution reaction over D-O-ZIS catalyst, (**c**) Two-dimensional contour plots of Raman vibration signals throughout the hydrogen evolution process, (**d**) Calculated Gibbs free energy of hydrogen adsorption (ΔG_H*_) at various active sites for ZIS, D-ZIS and D-O-ZIS, reproduced with permission from [112], Copyright 2024, Springer Nature.

In recent years, integrating theoretical calculations with multidimensional in situ characterization has emerged as an important direction in mechanistic studies of metal sulfide photocatalysis [62,97]. For example, Kelvin probe force microscopy (KPFM) can map changes in surface potential, whereas XAS provides information on local coordination environments and electronic structures [111]. Their combined use can offer a more comprehensive understanding of electronic behavior and structural evolution during photocatalysis. Nevertheless, evidence from a single characterization technique is generally insufficient to determine charge-transfer directions and operative reaction pathways with confidence. A multidimensional body of evidence should therefore be established by integrating structural analysis, spectroscopic characterization, electrochemical measurements, and theoretical calculations [62]. For instance, a decrease in PL intensity or EIS impedance alone does not conclusively establish a specific charge-transfer pathway; multiple complementary characterization methods are required to construct a more reliable mechanistic evidence base [109]. Combined characterizations and theoretical simulations are crucial to uncovering the photocatalytic mechanism of modified metal sulfides. Typical characterization results are summarized in Figure 2. Figure 2 assembles a suite of complementary spectroscopic experiments and theoretical calculations that exemplify multi-faceted mechanistic investigations for metal sulfide photocatalysts.

### 2.6. Photocorrosion and Intrinsic Stability Limitations

Despite their strong visible-light response and highly tunable electronic structures, metal sulfides remain susceptible to photocorrosion, metal leaching, and inadequate long-term stability, all of which substantially constrain their practical application [59,65]. CdS, in particular, is one of the most extensively investigated sulfides because of its strong reduction capability, yet it is also among the materials most severely affected by photocorrosion [55,65]. Under prolonged irradiation, photogenerated holes in CdS can oxidize lattice S^2-^ species, gradually converting them into oxidized sulfur species. This process is accompanied by Cd^2+^ release and disruption of the crystal structure, ultimately leading to a deterioration in catalytic performance. Suppressing hole accumulation and protecting the sulfide lattice are therefore central to improving the stability of CdS [65,66].

Photocorrosion is initiated when surface holes are not consumed rapidly enough by the intended oxidation reaction. They can oxidize lattice sulfide (S_2_^−^) to sulfur-containing products such as S0, polysulfides, or oxyanions, while charge compensation and bond loss promote metal dissolution and vacancy accumulation. The resulting under-coordinated sites may initially increase adsorption, but continued leaching can disrupt the lattice, alter band edges, expose secondary phases, and create nonradiative deep traps. In parallel, strongly reducing electrons may induce reductive surface reconstruction in some sulfides. Thus, activity loss can reflect several coupled processes rather than a single sulfur-oxidation step; operando spectroscopy, post-reaction elemental analysis, and dissolved-metal measurements are needed to identify them.

In complex aqueous environments, pH, inorganic ions, and organic contaminants can alter the surface state of a material and accelerate structural reconstruction or metal-ion leaching, thereby compromising its long-term catalytic performance [59,113]. Future evaluations of metal sulfide photocatalysts should therefore extend beyond initial activity and comprehensively consider long-term stability, environmental safety, and structural evolution under practically relevant operating conditions [62,114].

Building on the systematic discussion in this section of crystal and electronic structures, surface-active sites, charge-carrier dynamics, theoretical reaction descriptors, in situ characterization methods, and photocorrosion mechanisms, the following sections examine material-modification strategies and their extension to practical systems from two complementary perspectives: multidimensional structural regulation and photocatalytic applications across diverse scenarios. The intrinsic physicochemical properties of representative sulfides are consolidated in Table 1; subsequent discussions draw on these data to avoid repetitive descriptions of fundamental material characteristics.

## 3. Structural Regulation Strategies for Metal Sulfide Photocatalysts

**Figure 3:**
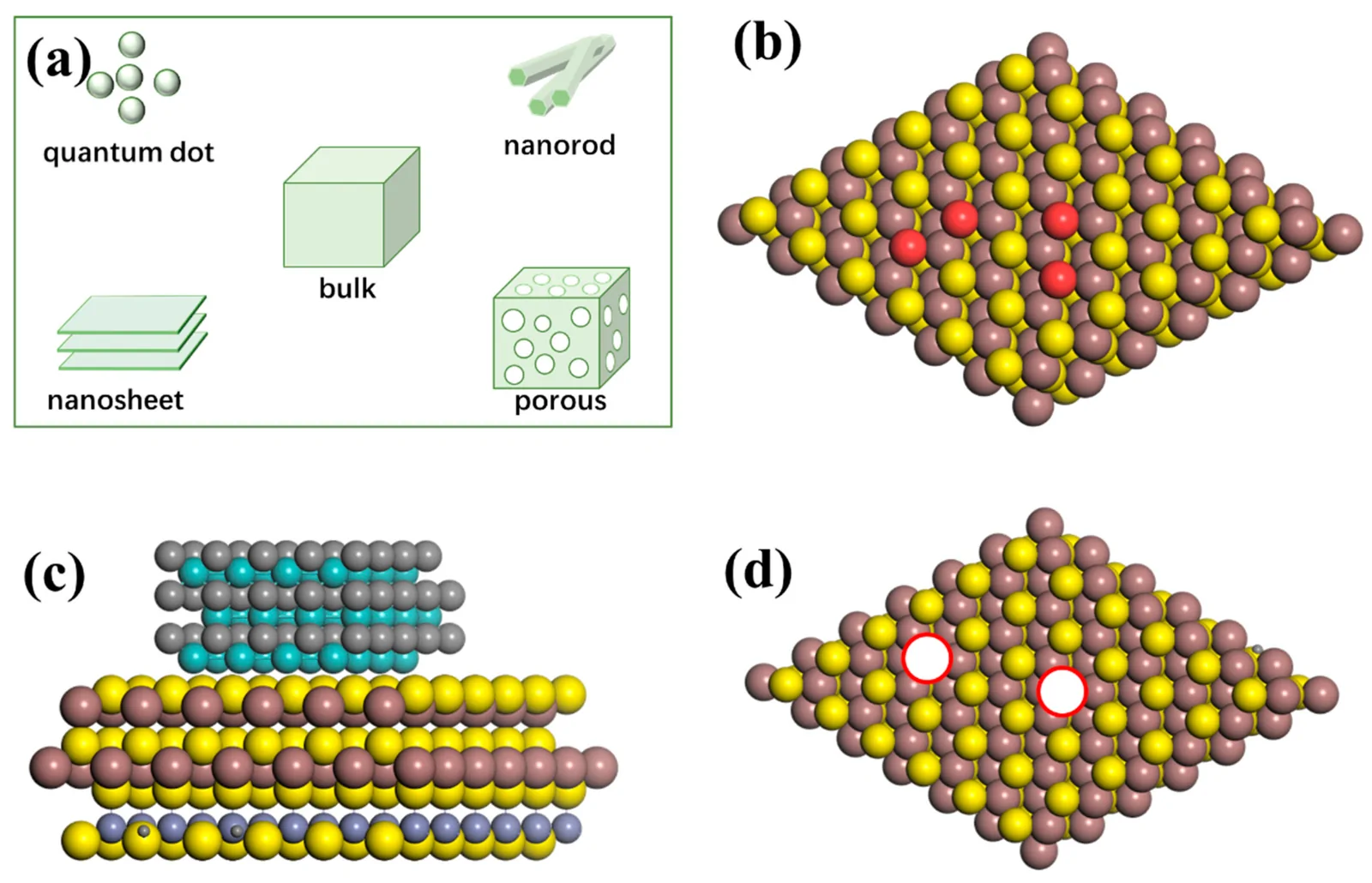
Schematic illustration of representative structural engineering strategies for metal sulfide photocatalysts: (**a**) morphology engineering, (**b**) aliovalent or isovalent atomic doping, (**c**) heterojunction construction between distinct phases, and (**d**) vacancy-type defect design. In panel (**b**), the substituted atoms occupy metal or sulfur lattice sites; panel (**c**) shows two compositionally distinct phases in contact; panel (**d**) denotes missing lattice atoms. The symbols are schematic and do not specify a particular element or crystal structure.

The photocatalytic performance of metal sulfides is governed not only by their intrinsic electronic structures but also by the combined effects of crystal structure, particle size, dimensionality, and the extent of surface exposure [38]. Structural engineering that regulates light absorption, charge-carrier transport, and surface reaction processes therefore represents an important approach to enhancing their photocatalytic performance. In recent years, extensive efforts have been devoted to morphology engineering, defect engineering, compositional regulation, interface engineering, and cocatalyst modification, with the aim of synergistically optimizing material structures and photocatalytic processes (Table 2). These structural regulation strategies do not operate independently; rather, each act on specific aspects of light absorption, charge transport, surface reactions, and material stability. Compared with optimization through an individual strategy, multidimensional and synergistic structural design has increasingly become a major direction for improving the overall performance of metal sulfides. Nevertheless, the individual strategies differ in their principal functions, and their effectiveness depends on how well they match the requirements of the target reaction and the intrinsic properties of the material [81]. Various structural engineering strategies have been exploited to improve the photocatalytic performance of metal sulfides. Five representative approaches are summarized in Figure 3.

### 3.1. Morphology Engineering: Regulation of Light Absorption and Mass Transport

Morphology engineering is one of the fundamental strategies for regulating nano-materials [115,116,117,118,119,120,121,122,123,124,125,126,127,128,129]. Controlling material size, dimensionality, and spatial architecture can modulate the specific surface area, exposed crystal facets, light-harvesting capability, and charge-carrier transport distance, thereby improving photocatalytic efficiency [73]. Compared with conventional particulate structures, one-dimensional (1D) nanorods and nanowires can provide more favorable electron-transport pathways and surface reaction regions without substantially altering the intrinsic electronic properties of the material [41,130]. CdS nanorods, for example, establish continuous 1D transport pathways that facilitate electron migration while retaining the intrinsic photoresponse of CdS. This architecture reduces charge losses during bulk transport and increases the probability that photogenerated electrons will reach the surface [41,55]. Relative to particulate CdS, such 1D morphologies favor directional electron transport and thus provide a more efficient supply of electrons for subsequent surface reactions.

**Figure 4:**
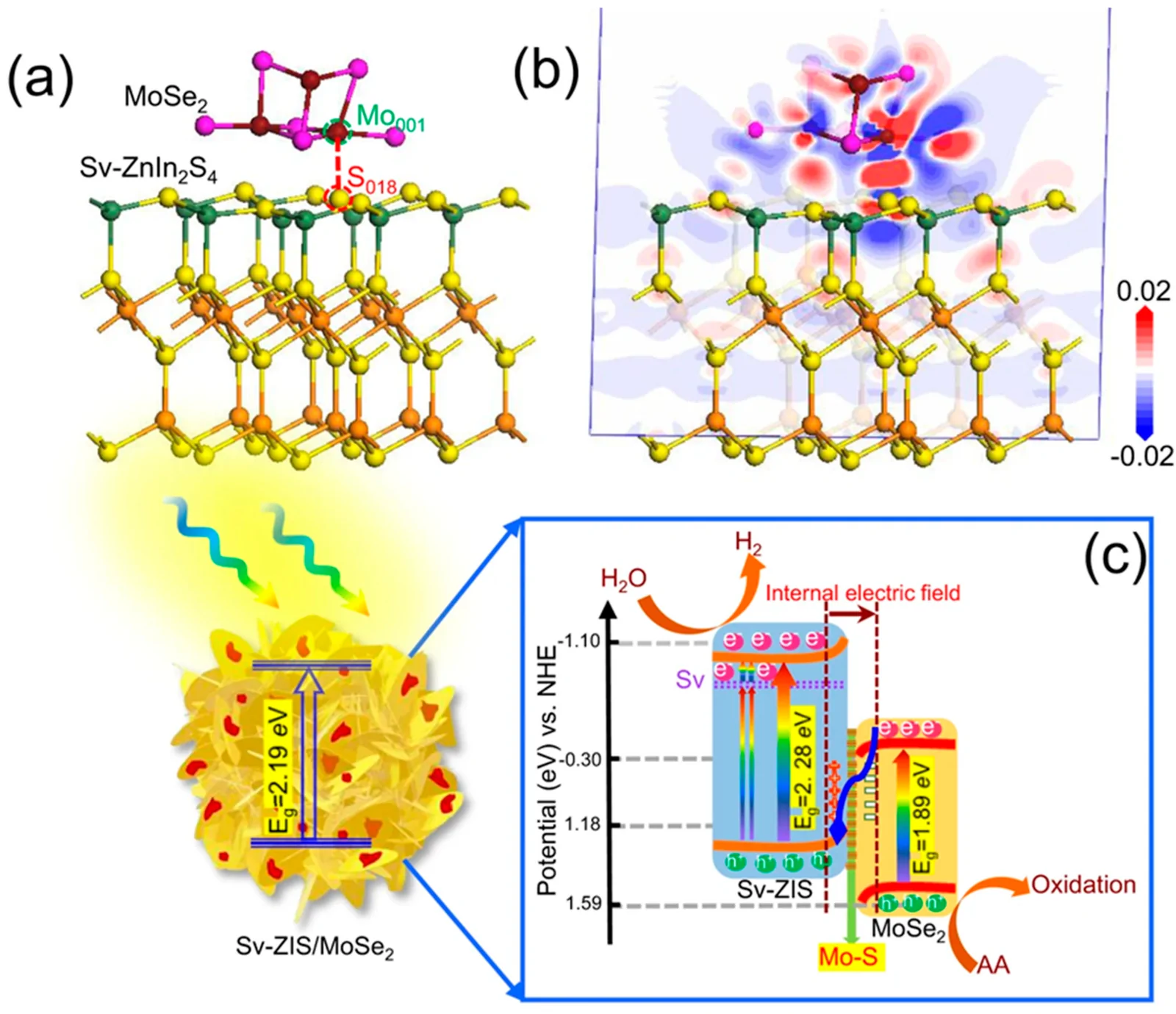
(**a**) Optimized geometric structure of the S_V_-ZnIn_2_S_4_/MoSe_2_ heterostructure, (**b**) Side-view charge density difference distribution of the S_V_-ZnIn_2_S_4_/MoSe_2_ heterostructure, (**c**) Photocatalytic reaction mechanism of the composite under visible light illumination, reproduced with permission from [131], Copyright 2021, Springer Nature.

Beyond conventional CdS nanorods, CdZnS solid solutions formed by varying the Cd/Zn ratio permit the morphology and electronic structure to be optimized concurrently [35]. For example, tetrapod-shaped Cd_0.9_Zn_0.1_S features branches extending in multiple directions, which enlarge the accessible surface reaction regions and improve light harvesting while providing a favorable structural platform for the subsequent construction of heterointerfaces. Two-dimensional (2D) layered structures similarly provide high surface exposure and tunable interfaces, making them well suited to further defect regulation and heterointerface construction [74]. Ultrathin 2D ZnIn_2_S_4_ nanosheets, for instance, expose a greater proportion of the surface and offer more reaction sites, while serving as stable structural platforms for subsequent defect and interface engineering [36,74]. Moreover, the relatively weak interlayer interactions in 2D materials facilitate exfoliation, defect introduction, and interfacial assembly, enabling further regulation of their electronic structures and surface properties [74]. Two-dimensional nanosheet substrates can also act as favorable platforms for growing secondary phases in situ to build heterostructures without destroying their intrinsic layered geometry. Representative synthetic pathways and morphological observations are given in Figure 4. MoSe_2_, for example, can be deposited on ZnIn_2_S_4_ nanoflowers, where the sheet-shaped morphology is well retained alongside abundant newly formed hetero-interfaces [131] (Figure 4a,b).

In addition to low-dimensional architectures, three-dimensional (3D) hierarchical and porous structures can also improve the photocatalytic performance of metal sulfides [132]. Hierarchical architectures are typically assembled from nanosheets, nanorods, or nanoparticles and can simultaneously provide a large specific surface area, enhanced light harvesting, and rapid mass transport [132]. Flower-like ZnIn_2_S_4_, for example, possesses an open 3D architecture that increases the utilization of incident light and facilitates the diffusion of reactant molecules across the catalyst surface [132,133]. Hollow architectures have also emerged as an important form of morphology regulation [134]. Their internal cavities promote multiple light reflections and shorten the distance over which photogenerated charge carriers must travel to reach the surface. Consequently, hollow metal sulfides commonly exhibit improved light utilization and more effective charge separation. The internal void space can additionally accommodate structural strain generated during photocatalysis, thereby improving cycling stability [135].

Morphology regulation alone, however, is generally insufficient to simultaneously suppress charge-carrier recombination and overcome sluggish surface reaction kinetics [81]. Morphology engineering is therefore commonly integrated with defect engineering, compositional regulation, and heterostructure construction to further optimize the electronic structure and reaction processes [38,40]. Combining a 2D architecture with defect engineering, for example, can increase the availability of surface reaction regions and tailor the interfacial electronic environment, enabling synergistic regulation of morphology and electronic structure [40,80]. Similarly, regulating the surface structure of CdS-based materials and introducing cocatalysts can enhance the utilization of photogenerated electrons and accelerate hydrogen-evolution kinetics [48,76].

Overall, morphology engineering improves photocatalytic performance primarily by regulating light-propagation pathways, molecular diffusion, and charge-carrier transport distances. One-dimensional structures are particularly advantageous for directional electron transport, whereas 2D structures favor the exposure of active interfaces and the construction of subsequently functionalized architectures. Three-dimensional hierarchical and hollow structures, by comparison, offer distinct advantages in light harvesting and mass transport. No universally optimal morphology therefore exists; an appropriate architecture should instead be selected according to the specific requirements of the target reaction for charge transport, surface adsorption, and material stability [38,81].

### 3.2. Defect Engineering: Tailoring Electronic Structures and Surface Reaction Sites

Building on the effects of defects on local electronic environments and surface reaction behavior discussed in Section 2.2, the emphasis of defect engineering has gradually shifted from the simple introduction of defects to their precise construction [136,137,138,139,140,141,142,143]. Current research therefore focuses on controlling defect type, concentration, and spatial distribution to optimize surface adsorption and reaction kinetics while balancing catalytic activity against structural stability [40,41]. Among the various defect types, sulfur vacancies (S vacancies) are regarded as some of the most common and important in metal sulfides because of their relatively low formation energies and pronounced ability to modify surface electronic structures [40,144]. In practice, the effects of these defects on catalytic performance depend strongly on their concentration and spatial distribution. An appropriate concentration of sulfur vacancies can optimize reactant-binding strength and interfacial electron transfer, whereas excessive defect formation may generate deep-level traps, increase the probability of electron–hole recombination, and compromise structural stability [145].

**Figure 5:**
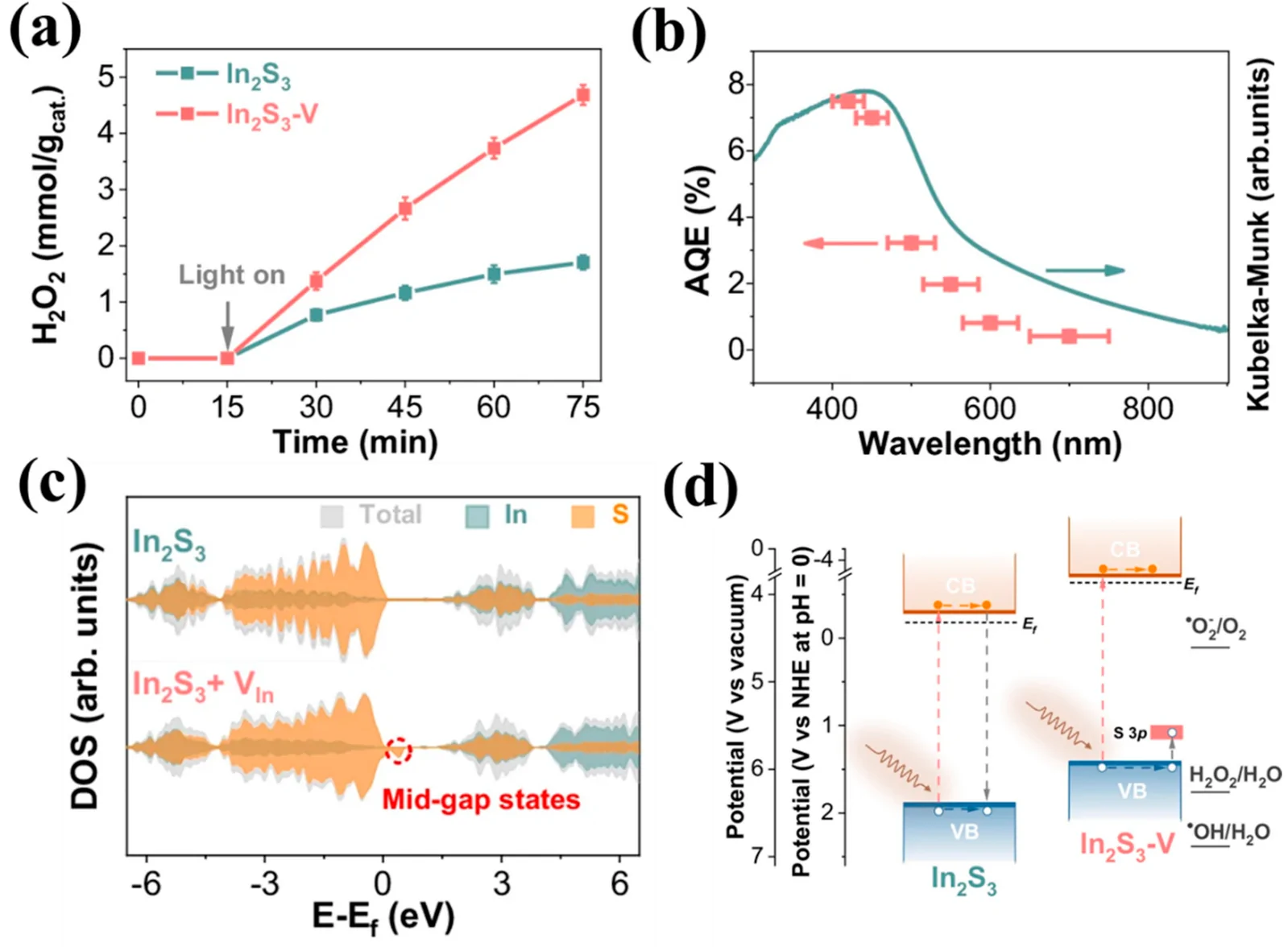
(**a**) Time-dependent photocatalytic H_2_O_2_ generation profiles of pristine In_2_S_3_ and defective In_2_S_3_-V samples, (**b**) Action spectra of In_2_S_3_-V for photocatalytic H_2_O_2_ production with UV–vis DRS spectra included for comparative analysis, (**c**) Density of states (DOS) calculated via DFT for pure In_2_S_3_ and V_In_-containing In_2_S_3_-V, where the mid-gap states induced by indium vacancies are highlighted with red dashed circles, (**d**) Schematic band alignment diagram illustrating the conduction band (CB) and valence band (VB) positions of pristine In_2_S_3_ and In_2_S_3_-V derived from UPS measurements, adapted with permission from Ref. [146] Copyright 2025, Springer Nature.

In the research from Zhu et al., surface indium vacancies (marked as V_In)_ were successfully implanted into In_2_S_3_ lattice, which substantially enhances the photocatalytic performance toward hydrogen peroxide synthesis, as shown in Figure 5 [146]. Under visible-light irradiation (λ ≥ 420 nm), the optimized catalyst achieves a prominent H_2_O_2_ production rate of 4.77 ± 0.05 mmol·h^−1^·g_cat_^−1^, along with an apparent quantum efficiency (AQE) of 7.49 ± 0.01% at the wavelength of 420 ± 20 nm. Mechanistic investigations demonstrate that the introduced V_In_ possesses multiple functional advantages in photocatalytic reactions. Specifically, In vacancies effectively improve the oxygen chemisorption capability of the catalyst, accumulate abundant photogenerated electrons to facilitate the oxygen reduction reaction (ORR), endow photogenerated electrons with strong reduction ability, and accelerate the desorption of generated H_2_O_2_, thereby synergistically promoting the photocatalytic H_2_O_2_ production process.

Structural comparisons between pristine and defective ZnIn_2_S_4_, various vacancy configurations, band-structure evolution, direct microscopic evidence and typical defect-forming routes. Theoretical computations show that sulfur vacancies rearrange atomic arrangements and modify the electronic band structure of ZnIn_2_S_4_ [147]. Aberration-corrected HRTEM offers straightforward experimental proof for intrinsic lattice vacancies Apart from sulfur vacancies, different vacancy sites such as In-faced S vacancies, Zn-faced S vacancies and metallic vacancies possess disparate formation energies [148]. Hydrothermal treatment, on the other hand, is capable of generating sulfur vacancies in the course of material preparation. Combined theoretical and experimental results stress that defect-related catalytic behavior strongly depends on vacancy types, atomic positions and formation energies.

In addition to promoting charge-carrier separation, sulfur vacancies can regulate the adsorption of reactant molecules on catalyst surfaces. Their specific role varies among reaction systems, although performance enhancement generally arises from the optimization of surface-binding strength and reaction kinetics. In HER systems, an appropriate defect concentration can improve H* adsorption. In CdS, for example, sulfur vacancies can modify the surface electronic environment and endow active sites with a more favorable H* adsorption strength, thereby enhancing hydrogen-evolution efficiency. For CO_2_ reduction and H_2_O_2_ production, defect regulation instead plays an important role in controlling the conversion of key intermediates. Sulfur vacancies in ZnIn_2_S_4_, for instance, can modulate the adsorption of oxygen species and facilitate O_2_ activation, thereby promoting the selective formation of H_2_O_2_. The principal advantage of defect engineering thus lies not in merely increasing the number of active sites but in optimizing specific reaction steps through control of the local electronic environment. Accordingly, different reactions require distinct defect types and concentrations: HER places greater emphasis on optimizing hydrogen adsorption, whereas CO_2_ reduction and H_2_O_2_ production depend more strongly on regulating intermediate adsorption.

Metal vacancies, in addition to sulfur vacancies, can effectively modify the local electronic structures of metal sulfides [40]. Because metal species participate directly in regulating the electronic properties of sulfide lattices, introducing metal vacancies can alter the Fermi-level position, charge-carrier concentration, and surface charge distribution [41]. For example, the formation of Zn or In vacancies in layered sulfides such as ZnIn_2_S_4_ can generate electron-deficient states, facilitate the transport of photogenerated electrons, and increase the number of surface-active sites [149].

Recent studies have further developed defect-concentration regulation strategies that employ thermal treatment, plasma treatment, chemical reduction, and in situ growth to achieve precise control over defect structures [150]. Mild sulfidation or treatment under a reducing atmosphere, for example, can introduce an appropriate concentration of sulfur vacancies while preserving the crystal structure, thereby avoiding the enhanced charge-carrier recombination associated with excessive defect formation [150]. The relationship between defect concentration and catalytic performance therefore commonly follows a volcano-type trend: a moderate defect concentration promotes the target reaction, whereas excessive defects may form deep-level traps, accelerate electron–hole recombination, and impair catalytic stability [40,65]. Defect engineering can also be integrated with other structural regulation strategies to further enhance the photocatalytic performance of metal sulfides [151]. Coupling defect regulation with heterostructure construction can improve interfacial charge transfer through defect-induced electronic modulation. Similarly, combining defects with cocatalysts can create additional electron-transfer pathways and optimize surface reaction kinetics [152].

Defect-engineered metal sulfide/carbon composites have also attracted increasing attention. Owing to their well-developed porous structures and abundant surface functional groups, agricultural-waste-derived carbon materials can promote the dispersion of sulfide particles, provide a stabilizing environment for defect structures, and improve electron transport and long-term cycling performance [60,153]. Nevertheless, defect engineering continues to face several challenges, including the difficulty of precisely controlling defect identity, complex defect-formation mechanisms, and insufficient defect stability during long-term operation [59]. In particular, an excessive concentration of sulfur vacancies under prolonged photocatalytic conditions may induce the migration of sulfur species and metal leaching, thereby accelerating photocorrosion [65,66]. In situ spectroscopic techniques, theoretical calculations, and kinetic analyses should therefore be integrated to establish a detailed understanding of the relationship between defect structures and catalytic performance [154], ultimately enabling the controlled construction and stable application of these defects [97,109].

Overall, defect engineering provides an important link between the electronic structure of a material and its surface reaction processes, but the resulting performance enhancement depends on precise control over defect type, concentration, and spatial distribution. Future defect design should move beyond the simplistic pursuit of maximizing defect density. Instead, reaction-oriented methods for defect construction should be developed and synergistically integrated with interface engineering, compositional regulation, and other complementary strategies [151].

### 3.3. Composition Engineering: Optimization of Band Structures and Catalytic Selectivity

**Figure 6:**
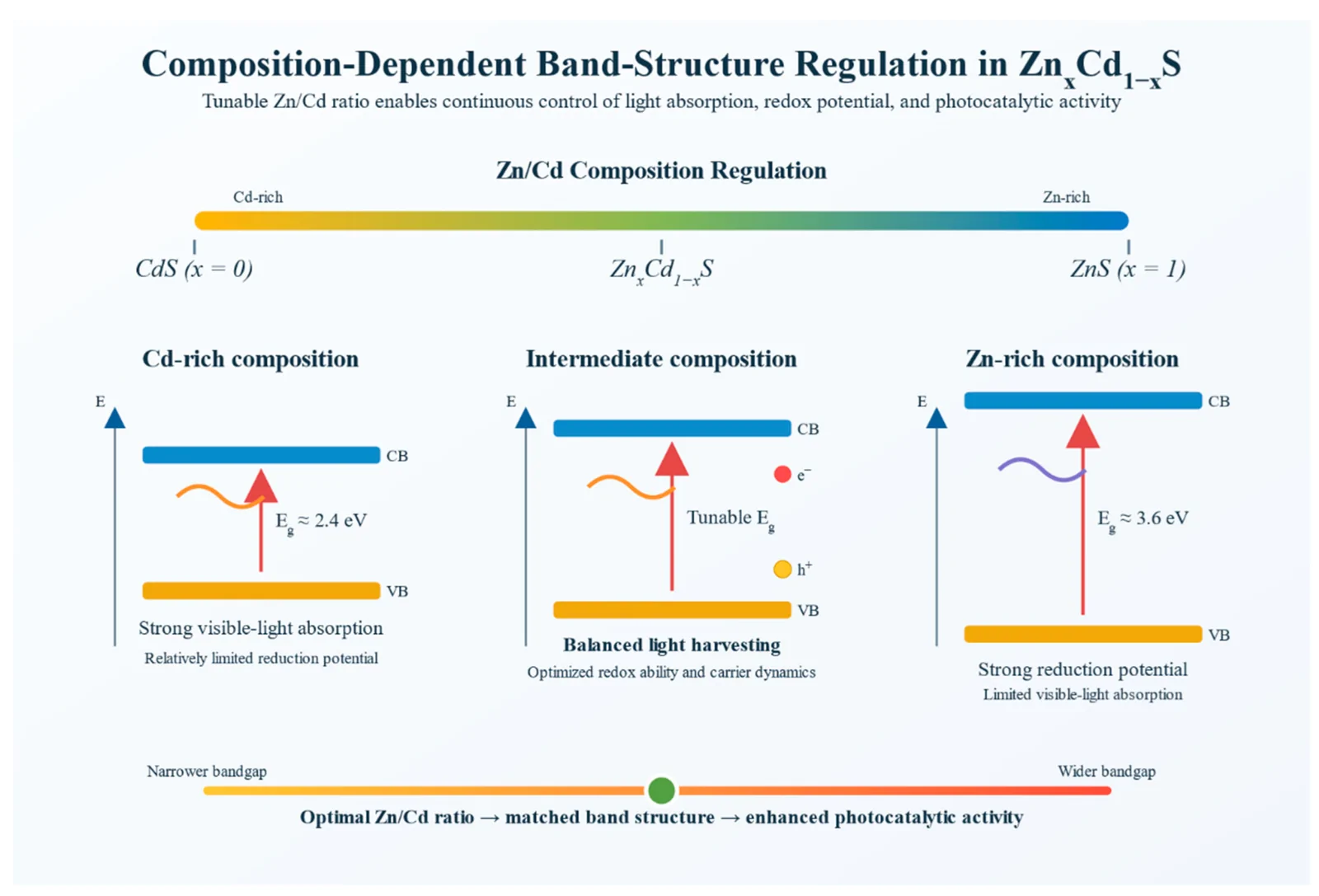
Composition-Dependent Band-Structure Regulation in Zn_x_Cd_1−x_S.

As shown in Table 1, different metal sulfide systems exhibit pronounced variations in their band structures and electronic characteristics. To overcome the limitations of single-component materials in spectral response, stability, and compatibility with specific reactions, composition engineering enables the targeted optimization of material properties by adjusting elemental ratios and interelement electronic coupling [155,156,157,158,159,160,161,162,163,164,165]. Introducing different metal elements or varying their relative proportions can alter the crystal structure, electronic-state distribution, bandgap, and surface reaction characteristics [166]. These changes allow light absorption, charge-carrier transport, and catalytic activity to be optimized synergistically [73,162,167]. Compared with single-component, multinary sulfides and solid-solution materials provide greater flexibility for band-structure regulation through electronic coupling among their constituent elements [168]. For example, As depicted in Figure 6, ZnCdS solid solutions formed by varying the Cd/Zn ratio exhibit continuously tunable bandgaps and electronic structures, allowing their properties to be matched to the requirements of a target reaction [169,170,171,172,173,174]. ZnCdS thus offers a broader design space than its single-component counterparts for regulating spectral response, charge-carrier transport, and surface reaction processes. Complementary DFT calculations further interpret the underlying structural and electronic origin of this tunability, where crystal configurations and band structures evolve systematically with changing Zn/Cd atomic fractions [175].

The effect of doping depends on the host lattice and the dopant’s valence, ionic radius, electronegativity, and preferred coordination. Substitution can shift the band edges or Fermi level, introduce shallow donor/acceptor states, and change carrier density; at higher loading it may instead generate deep traps, strain, or secondary phases. In layered ZnIn_2_S_4_, for example, substitution at Zn and In sites is not electronically equivalent because the two cations have different coordination and contributions to the band edges. Dopant identity and site occupancy should therefore be verified rather than inferred from precursor composition alone. X-ray absorption or atomic-resolution microscopy, together with charge-density calculations and concentration-dependent activity, can distinguish a genuine lattice-doping effect from surface decoration or phase segregation.

Composition regulation in ZnCdS can balance the strong reduction capability of CdS against the higher stability of ZnS [169]. As the Zn content increases, the bandgap gradually widens and the light-absorption range shifts, accompanied by changes in the electronic structure and charge-carrier transport behavior. Optimizing the Zn/Cd ratio can therefore establish an appropriate balance among light harvesting, charge-separation efficiency, and surface reactivity. In addition to ZnCdS solid solutions, Cd-free multinary sulfides such as ZnIn_2_S_4_ have become an important research focus. Regulation of the Zn/In ratio and overall elemental composition can further optimize the electronic structure and distribution of surface-active sites, thereby improving the suitability of ZnIn_2_S_4_ for different photocatalytic processes [176,177,178,179]. Synergistic interactions among Zn, In, and S regulate its electronic structure, allowing ZnIn_2_S_4_ to combine a favorable visible-light response with relatively high stability [180,181,182,183,184]. Compared with morphology and defect engineering, a principal advantage of composition engineering is the broader scope it provides for electronic-structure regulation. Optimization of elemental ratios and exploitation of multimetal synergy allow the spectral response, redox capability, and surface reaction selectivity to be tailored to specific reaction requirements. Increasing compositional complexity, however, does not necessarily improve performance. Complex elemental systems may introduce structural instability and obscure the origin of catalytic activity; precise composition design must therefore be supported by theoretical calculations and experimental validation.

Further adjustment of the Zn/In ratio can alter the surface electron density and spatial distribution of active sites, thereby influencing photocatalytic reaction processes [185]. Other multinary sulfides, including In_2_S_3_, SnS_2_, Bi_2_S_3_, and CuInS_2_, likewise offer considerable potential for composition regulation [36,186]. In_2_S_2_ combines favorable light absorption with good stability, while elemental regulation or defect introduction can further improve its charge-carrier transport properties [186]. Layered sulfides such as SnS_2_ and Bi_2_S_3_ have attracted attention in pollutant degradation and photoelectric conversion because of their relatively narrow bandgaps and diverse surface chemistry [187]. Copper-based sulfides such as CuInS_2_, meanwhile, exhibit strong visible-light absorption and multielectron-transfer characteristics, giving them potential for CO_2_ reduction and solar-fuel production [187,188].

In addition to regulating the host composition, elemental doping is an important means of improving the performance of materials [147,189]. The incorporation of transition metals, rare-earth elements, or nonmetallic elements can modify the local electronic structure, introduce new electronic states, and regulate charge-carrier concentration and transport. Doping with transition metals such as Ni, Co, and Mo, for example, can optimize the surface electronic environment of metal sulfides, improve electron-transfer efficiency, and facilitate proton reduction during HER [48,105]. Transition-metal doping can cooperate with intrinsic sulfur vacancies to tune the photocatalytic properties of layered sulfides. In the case of Cu-modified SnS_2−x_, solvothermal-hydrothermal treatment produces flower-shaped Cu/SnS_2−x_ architectures that simultaneously contain Cu dopants and sulfur-vacancy defects. Under visible-light illumination, the in situ-formed defect states serve as electron traps to speed up charge separation and facilitate the water-reduction hydrogen-evolution reaction. Integrating heteroatom doping with native defects offers a practical way to optimize electronic configurations as well as surface reaction kinetics. Heteroatom doping can also modify surface adsorption properties and reaction selectivity [147]. Dopant species may regulate the adsorption strength of CO_2_ and the binding of key intermediates, thereby altering the product distribution of CO_2_ reduction. Meanwhile, doping-induced changes in electronic structure can facilitate the activation of oxygen species and improve the efficiency of H_2_O_2_ production and selective oxidation reactions [77,106].

In recent years, composition engineering has evolved from simple elemental substitution toward the synergistic regulation of multiple elements [190]. Multimetal sulfides, for example, exploit electronic coupling among different metals to simultaneously optimize the band structure, surface-active sites, and reaction kinetics [104,190]. Compared with conventional single-component materials, multicomponent sulfides offer a richer parameter space for electronic regulation and provide new opportunities for designing efficient photocatalytic systems [190]. Nevertheless, composition engineering still faces challenges associated with precise control over elemental ratios, structural instability caused by complex elemental incorporation, and difficulty in identifying the origin of catalytic activity [59]. Theoretical calculations should therefore be integrated with advanced characterization techniques to elucidate how elemental regulation affects electronic structures, interfacial behavior, and reaction pathways, ultimately enabling the precise design of compositions according to target-reaction requirements [95,97].

Overall, composition engineering is particularly valuable for addressing the difficulty of simultaneously optimizing spectral response, stability, and reaction selectivity in single-component materials. Future research is expected to progress from simple elemental substitution toward synergistic multielement regulation. Machine learning and high-throughput computational methods may further enable predictive relationships among composition, structure, and performance to be established [151].

### 3.4. Interface Engineering: Construction of Heterojunctions for Directional Charge Transfer

As discussed in Section 2.3, photogenerated charge carriers undergo substantial recombination losses during transport. The construction of heterointerfaces can mitigate these losses by exploiting interfacial interactions to regulate charge-transfer behavior [191,192,193,194,195,196,197,198,199,200,201,202]. This section discusses the principal types of interface architectures and the performance improvements afforded by their construction.

Based on interfacial band alignment and charge-transfer pathways, heterostructures can be broadly classified as p–n junction, type-II, Z-scheme, and S-scheme heterojunctions, as well as Schottky junctions [203,204,205,206,207,208,209,210,211,212,213,214,215]. In a conventional type-II heterostructure, favorable band alignment drives electrons from the conduction band of one semiconductor to that of the other while promoting the spatial separation of electrons and holes [216]. This configuration, however, often sacrifices redox capability because electrons and holes with higher reduction and oxidation potentials, respectively, are consumed during charge redistribution, potentially weakening the thermodynamic driving force for photocatalytic reactions. Z-scheme heterostructures have consequently attracted considerable attention as a means of overcoming the insufficient redox capability of conventional type-II systems [217]. By mimicking the electron-transfer process of natural photosynthesis, a Z-scheme system selectively recombines charge carriers with relatively weak redox capabilities while retaining highly reducing electrons and strongly oxidizing holes. It can therefore combine efficient charge separation with strong redox capability [217]. No heterojunction architecture is universally superior, however. Type-II systems generally offer relatively straightforward interface construction, whereas Z-scheme and S-scheme systems are better able to preserve strong redox potentials. The key to heterostructure design is therefore not the selection of a particular theoretical model, but the optimization of interfacial contact quality, charge-transfer direction, and operational stability according to the requirements of the target reaction.

In recent years, CdS/ZnS, CdS/ZnIn_2_S_4_, and ZnIn_2_S_4_-based composites have been widely investigated for constructing efficient heterointerfaces. Optimization of the interfacial contact configuration and component ratios can improve charge-carrier utilization while maintaining strong redox capability [217,218]. Compared with the conventional Z-scheme model, the S-scheme description explicitly considers Fermi-level equilibration, band bending, and built-in electric-field formation upon interfacial contact, thereby providing a more detailed account of charge-transfer behavior across semiconductor interfaces [219]. Because Z-scheme and S-scheme heterostructures better reconcile charge-separation efficiency with redox capability than conventional type-II systems, they have become important directions in the interface engineering of metal sulfides [219,220].

S-scheme interface engineering has been widely applied to metal sulfide systems based on CdS, ZnIn_2_S_4_, and ZnCdS [219]. In a CdS/ZnIn_2_S_4_ heterostructure, for example, interfacial charge redistribution and the resulting built-in electric field facilitate the transport of photogenerated electrons while preserving a strong reduction capability [221,222]. For Cd-based sulfides with limited stability, core–shell interfaces can additionally reduce the risk of photocorrosion by providing a protective surface layer. In CdS/ZnS structures, a ZnS shell improves the surface stability of CdS while maintaining effective charge transfer [66,105,110]. In addition to semiconductor–semiconductor heterojunctions, metal–semiconductor Schottky junctions represent another important interface strategy for enhancing the catalytic performance of metal sulfides [66]. When metal nanoparticles contact a sulfide semiconductor, the difference in their work functions produces an interfacial potential barrier. This barrier can promote the migration of photogenerated electrons toward the metal sites and suppress electron–hole recombination. The metal sites may simultaneously serve as surface reaction centers, lower kinetic barriers, and improve the utilization of photogenerated electrons [48,66].

**Figure 7:**
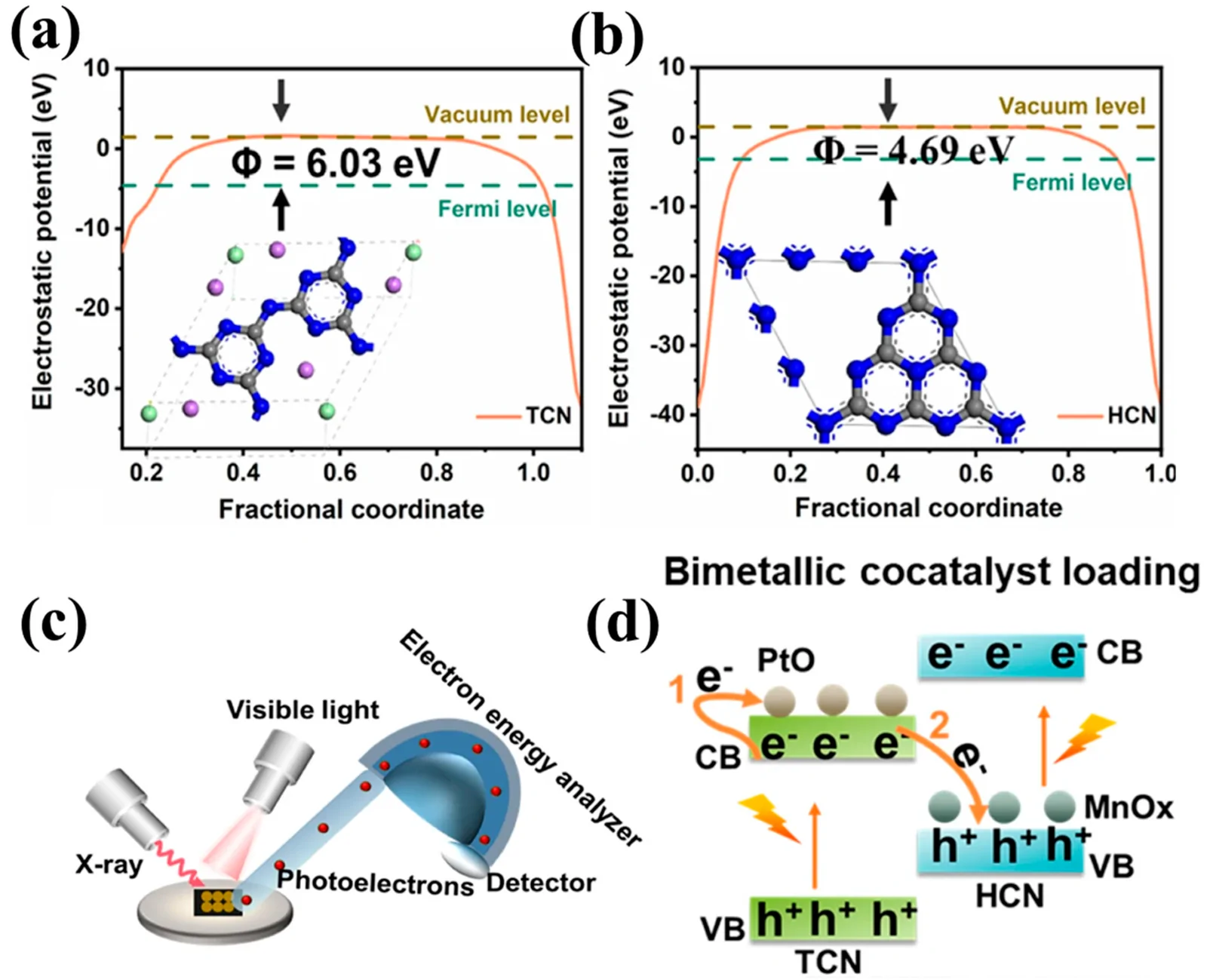
(**a**) Work function distributions of TCN, and (**b**) HCN (insets display the top-view slab models of corresponding samples), (**c**) Schematic setup of in situ XPS measurements with a xenon lamp serving as the illumination source, (**d**) Photogenerated electron transfer mechanism at the interface of the TH1:4 composite modified with bimetallic cocatalysts, adapted with permission from Ref. [224] Copyright 2023, Springer Nature.

Defect-assisted interface engineering has also attracted growing attention. Introducing defects such as sulfur vacancies near a heterointerface can modify the local electronic structure, strengthen interfacial electronic coupling, and facilitate the adsorption and activation of reactant molecules [151,223]. Defect-engineered CdS/ZnS and ZnIn_2_S_4_-based heterostructures, for example, can combine defect-induced electronic modulation with interfacial electric-field effects to achieve more efficient charge-carrier transport [105,153]. Nevertheless, interface engineering continues to face difficulties in precisely controlling interfacial structures, resolving controversies surrounding interfacial mechanisms, and maintaining long-term stability [59]. In some proposed S-scheme systems, in particular, work-function measurements, changes in PL signals, or EIS results alone are insufficient to establish the direction of charge transfer. Comprehensive verification using complementary methods—including Kelvin probe force microscopy (KPFM), in situ X-ray photoelectron spectroscopy (XPS), X-ray absorption spectroscopy (XAS), and theoretical calculations—is therefore required [97,106,220]. Experimental evidence for built-in electric-field formation and S-scheme charge transfer at heterointerfaces can be obtained by combining KPFM measurements with theoretical calculations, as illustrated in Figure 7. KPFM measurements record surface-potential variations of triazine/heptazine crystalline carbon nitride under dark and light conditions, offering experimental proof of interfacial electric-field effects originating from Fermi-level equilibration Schematic band-alignment diagrams further visualize band bending, built-in electric-field generation and the complete S-scheme photocatalytic reaction pathway.

Overall, interface engineering is among the most effective strategies for enhancing the photocatalytic performance of metal sulfides because it can simultaneously regulate charge separation and surface reactivity. As heterostructures become increasingly complex, however, future research should progress beyond the simple construction of heterointerfaces toward precise control of interfacial structures, rigorous identification of operative charge-transfer pathways, and improved long-term stability [41,48,132,151].

### 3.5. Cocatalyst and Single-Atom Engineering: Acceleration of Surface Reaction Kinetics

Building on the principles of reaction-barrier regulation discussed in Section 2.4, cocatalyst engineering enhances surface reaction kinetics primarily by creating new active centers and optimizing interfacial electronic coupling [15,225,226,227,228,229,230,231].

As a representative bimetallic boride cocatalyst, NiCoB offers distinct advantages in regulating interfacial electronic coupling and active-site properties. When coupled with CdS or CdZnS, NiCoB can redistribute interfacial electron density and improve HER kinetics by modifying the properties of the active sites [232,233]. In a Cd_0.9_Zn_0.1_S/NiCoB Schottky heterostructure, for example, NiCoB nanoparticles form intimate interfacial contact with CdZnS. Owing to the difference in their work functions, photogenerated electrons migrate directionally from CdZnS to NiCoB, inducing interfacial charge redistribution and promoting the spatial separation of charge carriers [54,56]. Theoretical calculations further indicate that ΔG_H*_ at the key active sites is optimized, establishing a more favorable balance between hydrogen adsorption and desorption and thereby accelerating hydrogen-evolution kinetics. The system achieves a hydrogen evolution rate of 108.39 mmol g^−1^ h^−1^, demonstrating the ability of bimetallic boride cocatalysts to regulate interfacial electronic structures and optimize HER pathways. In the NiCoB/CdS system, NiCoB additionally exhibits a favorable photothermal response and magnetic recoverability. Its function therefore extends beyond conventional promotion of electron transfer to encompass photothermal synergy and catalyst recycling, offering a new design strategy for the engineering application of non-noble-metal cocatalysts [56].

In addition to metal borides, transition-metal sulfide cocatalysts such as MoS_2_, NiS, and CoS have been widely incorporated into metal sulfide photocatalytic systems [35,37]. By establishing electronic coupling across semiconductor/cocatalyst interfaces, these materials provide reaction sites with low energy barriers and improve the utilization of photogenerated electrons during HER [104,234]. Single-atom cocatalysts can further increase active-site utilization relative to conventional nanoscale cocatalysts, although their practical implementation remains constrained by atomic stability, control over loading levels, and the difficulty of identifying the actual active structures. Future cocatalyst design should therefore progress from merely improving electron-transfer efficiency toward precise regulation of interfacial reaction pathways.

**Figure 8:**
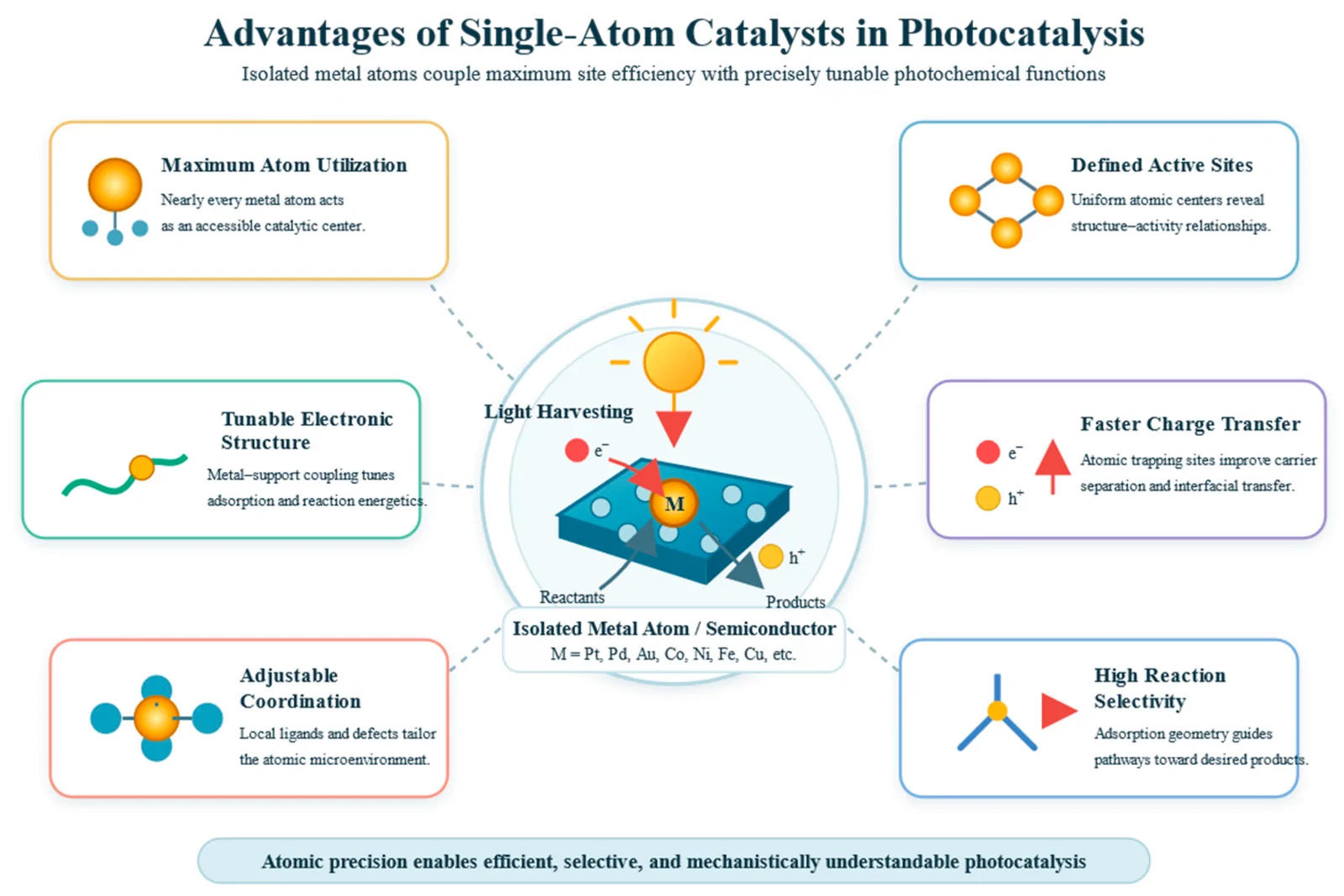
The advantages of single-atom catalysts in photocatalysis.

As shown in Figure 8, single-atom cocatalysts have attracted increasing attention in catalytic systems because of their high atom-utilization efficiency, well-defined electronic structures, and high density of exposed active sites [235,236,237,238,239]. Compared with conventional nanoparticulate cocatalysts, isolated atomic sites provide more uniform active centers and regulate interfacial charge transfer through strong metal–support interactions [240]. In a Pd single-atom/CdS system, for example, isolated Pd sites coordinated by sulfur promote directional interfacial electron migration. These sites also function as electron-transfer centers, accelerating the transfer of photogenerated electrons from CdS to the active sites and consequently improving charge-carrier separation and photocatalytic reaction kinetics [235]. Aberration-corrected STEM and synchrotron-based X-ray absorption spectroscopy represents powerful characterization tools for probing single-atom cocatalysts, which is exemplified by the Co-sZIS system. HAADF-STEM micrographs directly show well-dispersed isolated Co single atoms anchored on the ZnIn_2_S_4_ support. XANES and FT-EXAFS data shed light on the electronic state and local coordination environment of Co sites, and EXAFS fitting rules out the existence of metallic Co-Co bonds. Complementary wavelet-transform EXAFS contour plots further identify dominant Co-O coordination and confirm the atomic nature of Co species. For sulfide-supported single-atom photocatalysts, this set of multi-technique characterizations is essential to distinguish genuine single-atom sites from metal clusters or nanoparticles [241,242,243].

The role of cocatalysts has gradually evolved from passive electron trapping toward the active regulation of interfacial electronic structures. Early cocatalysts such as Pt and MoS_2_ were used primarily to improve electron utilization, whereas recently developed bimetallic borides, sulfides, and single-atom sites can additionally optimize catalytic pathways by regulating local electronic structures and the adsorption of reaction intermediates. Increasing cocatalyst loading indiscriminately should nevertheless be avoided because excessive loading may reduce light absorption, mask active sites, and compromise control over the interfacial structure. Future studies should integrate in situ characterization with theoretical calculations to identify the actual active structures of cocatalysts and clarify their roles under dynamic reaction conditions [97,106].

Overall, cocatalyst engineering primarily addresses sluggish surface reaction kinetics in photocatalytic processes. Its role has expanded from electron trapping to the coordinated regulation of interfacial electronic structures and reaction pathways. Compared with noble-metal systems, non-noble-metal and single-atom cocatalysts are expected to assume a more prominent role in balancing catalytic performance, cost, and scalability.

### 3.6. Synergistic Regulation Strategies: Integration of Multiple Structural Functions

As research has advanced, individual structural regulation strategies have become increasingly inadequate for meeting the demands of complex photocatalytic reactions. The synergistic integration of multiple strategies has therefore emerged as an important direction in the design of metal sulfides. Different forms of structural engineering perform complementary functions: defect engineering can tailor surface electronic structures, heterojunctions can facilitate charge-carrier transport, and cocatalysts can lower surface reaction barriers [244,245,246]. Multiscale structural design can integrate these functions to simultaneously improve light absorption, charge separation, interfacial transfer, and surface reaction kinetics, thereby overcoming performance limitations inherent to individual regulation strategies.

For example, coupling defect engineering with heterojunction construction can concurrently optimize interfacial charge transfer and catalytically active sites [186]. In defect-engineered ZnIn_2_S_4_-based S-scheme systems, sulfur-vacancy-induced electronic modulation acts in concert with the interfacial built-in electric field, improving photogenerated charge-carrier separation and accelerating surface reaction kinetics [186,234]. Similarly, introducing defect structures into CdS-based systems in combination with Ni- or Co-based cocatalysts can enhance both electron transport and surface hydrogen-evolution kinetics, enabling the coordinated optimization of charge transfer and catalytic reactions [55,56]. The synergistic integration of multicomponent sulfides, heterointerfaces, and carbon-based supports has also attracted growing attention [219]. Coupling metal sulfides with conductive carbon materials, for instance, can improve catalyst dispersion, promote interfacial electron transfer, and enhance cycling stability [247].

Agricultural-waste-derived carbon materials have recently been employed as composite supports for metal sulfides because of their well-developed porous structures, abundant surface functional groups, and tunable electrical conductivity [56,248]. These carbon supports primarily contribute to catalyst immobilization, enhanced electron transport, and pollutant enrichment, offering a promising route for extending metal sulfide photocatalytic systems to complex aqueous environments. Nevertheless, coupling multiple strategies does not mean that greater structural complexity necessarily yields better performance [62]. Excessive complexity may increase the difficulty of material synthesis, compromise structural stability, and obscure the actual origin of catalytic activity [133]. In systems containing multiple interfaces and components, different structural factors may also exert competing effects. In situ characterization, theoretical calculations, and kinetic analyses are therefore required to distinguish the contributions of individual structural units [109,132]. Future structural design should consequently focus on elucidating and exploiting the synergistic mechanisms among different regulation strategies rather than merely combining them [105,185].

## 4. Energy, Environmental, and Monitoring Applications of Metal Sulfide Photocatalysts

Metal sulfides have been extensively investigated for solar-driven hydrogen production and other applications because of their tunable electronic structures, strong visible-light response, and abundant surface-active sites (Table 3) [48]. However, different hydrogen evolution reaction (HER) systems impose distinct requirements on catalyst electronic structures, charge-carrier transport, and surface reaction sites. Consequently, research emphasis has gradually shifted from simply increasing hydrogen evolution rates toward structure-guided optimization of electron transport and reaction kinetics [48,76].

### 4.1. Hydrogen Evolution Reaction and Biomass-Assisted Hydrogen Production

Hydrogen evolution reaction is one of the earliest and most extensively studied applications of metal sulfides [249,250,251,252,253,254,255,256,257,258]. Most metal sulfides possess relatively narrow bandgaps and sufficiently negative conduction-band positions, enabling them to generate strongly reducing electrons under visible-light irradiation [259]. They have therefore attracted sustained attention for solar-driven hydrogen production.

Many reported HER rates are obtained with sacrificial electron donors, which rapidly consume photogenerated holes and suppress sulfide photocorrosion. Such measurements establish reduction activity under donor-assisted conditions but do not demonstrate overall water splitting or a self-sustained solar-to-hydrogen process [260]. Donor identity, concentration, pH, and oxidation products should be reported because they affect charge balance and apparent rates. Reducing donor dependence requires coupling proton reduction to a viable oxidation half-reaction, for example, water oxidation or selective biomass upgrading, and demonstrating product stoichiometry, carbon balance where relevant, and sustained operation without an externally added hole scavenger. Systems using biomass-derived donors can improve the value proposition but should be described as photoreforming rather than overall water splitting.

CdS is a representative visible-light-responsive sulfide semiconductor whose conduction-band position satisfies the thermodynamic requirement for H^+^ reduction, and it has long served as an important model material for HER studies [261,262,263]. Nevertheless, CdS suffers from severe photogenerated charge-carrier recombination and photocorrosion. Under prolonged irradiation, oxidation of lattice sulfur and the release of Cd^2+^ can disrupt the crystal structure, limiting long-term operational stability [264,265,266,267,268]. Early studies demonstrated that loading electron-trapping cocatalysts such as Pt could accelerate hydrogen-evolution kinetics on CdS surfaces and improve the utilization of photogenerated electrons [235]. The high cost of noble metals, however, limits their large-scale application. Recent research has therefore increasingly focused on non-noble-metal cocatalysts based on Ni, Co, and Mo to balance catalytic activity against cost [269,270,271,272].

Defect regulation, heterostructure construction, and cocatalyst modification have been widely employed to improve the performance of CdS-based systems [49,50]. For example, forming solid solutions between CdS and ZnS can regulate the electronic structure and improve photostability. Continuous composition regulation in Zn_x_Cd_1-x_ZnS enables the spectral response and reduction capability to be tuned, resulting in high activity for visible-light-driven hydrogen evolution [41,51]. By varying the Zn/Cd ratio, the bandgap and conduction-band position of ZnCdS solid solutions can be continuously adjusted. This tunability enables a more favorable balance among light absorption, charge-carrier transport, and reduction capability, making ZnCdS an important material platform for efficient HER systems.

Cd-free, two-dimensional layered ZnIn_2_S_4_ has also emerged as an important platform for HER because of its favorable light absorption and structural tunability [273]. Constructing S-scheme heterostructures, introducing defects, and loading cocatalysts can facilitate interfacial electron transfer and improve hydrogen-evolution efficiency [50,220]. Sulfur vacancies can regulate the surface electron distribution and strengthen proton adsorption, whereas heterointerfaces can reduce charge-carrier recombination losses. Together, these effects enable the coordinated optimization of electron transport and surface reaction kinetics [49,50]. For instance, the internal electric field in a ZnIn_2_S_4_/FePO_4_ S-scheme heterostructure promotes directional electron transfer and improves photogenerated charge-carrier separation, thereby enhancing HER performance [220].

**Figure 9:**
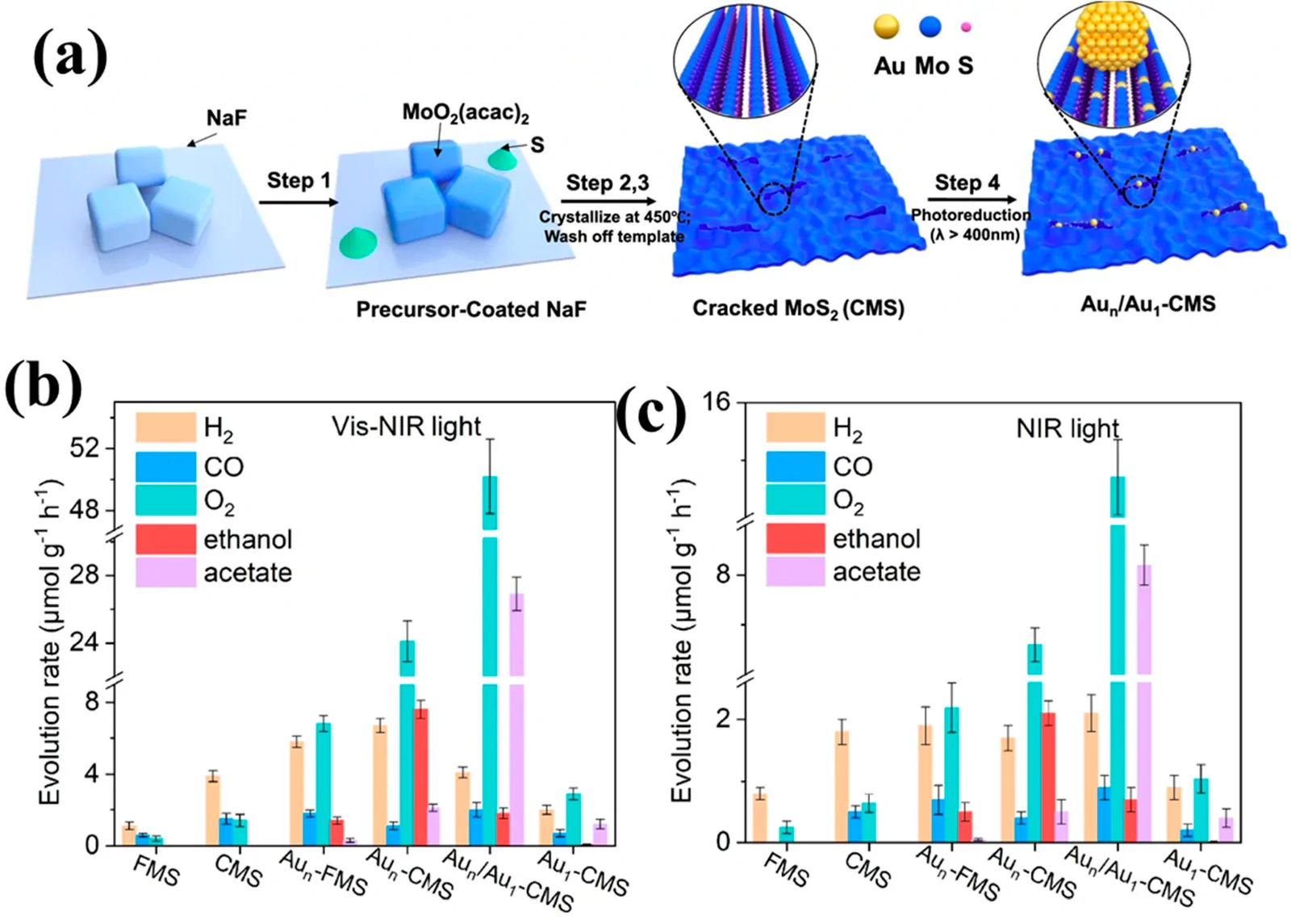
(**a**) Schematic diagram for the synthetic procedure of Au_n_/Au_1_-CMS composites, (**b**) Generation rates of products over Au_n_/Au_1_-CMS under Vis-NIR irradiation, and (**c**) Product evolution profiles under sole NIR illumination, adapted with permission from Ref. [274], Copyright 2024, Springer Nature.

Representative CO_2_ performances single atom Au MoS_2_ are compiled in Figure 9. Activity comparisons among various ZnIn_2_S_4_-derived composites demonstrate the performance improvement brought about by heterostructure construction (Figure 9) [274]. Activity can be further enhanced by combining defect engineering with non-noble-metal cocatalyst modification, as seen in sulfur-vacancy-containing Cu-modified SnS_2-x_. Time-resolved hydrogen-evolution profiles and Cu-content gradient tests pinpoint 5% Cu/SnS_2-x_ as the optimal catalyst within this material series (Figure 9b,c). The accompanying mechanistic scheme shows that sulfur-vacancy-derived defect states trap photogenerated carriers, whereas Cu sites function as active centers to accelerate water-reduction kinetics. These cases illustrate that coupling defect regulation and cocatalyst decoration constitutes an effective strategy to achieve high HER activity for metal sulfides [259].

Similarly, hierarchical heterostructures constructed from CdZnS and Cu-based sulfides can enhance both light absorption and surface reaction kinetics, enabling efficient hydrogen evolution under visible light [93]. The development of non-noble-metal cocatalysts has further improved the HER performance of metal sulfides [55]. In addition to conventional Pt cocatalysts, Ni-, Co-, and Mo-based materials can provide abundant active sites and optimize H* adsorption [275]. In NiCoB-modified CdZnS, for example, a Schottky heterointerface facilitates electron transfer while optimizing the Gibbs free energy of hydrogen adsorption, resulting in efficient hydrogen evolution [56]. In NiCoB/CdS systems, the bimetallic boride cocatalyst regulates the interfacial electronic structure and surface-active sites, further improving electron transport and hydrogen-intermediate adsorption. This approach provides a new direction for the design of non-noble-metal cocatalysts.

Most current HER studies, however, remain focused on half-reactions conducted in the presence of sacrificial reagents. Practical solar hydrogen production continues to be constrained by quantum efficiency, long-term stability, and reactor scale-up [67,276]. Future studies should therefore devote greater attention to sacrificial-agent-free systems, realistic aqueous environments, and continuous hydrogen-production processes [67,277].

Beyond conventional hydrogen production from water splitting, biomass-assisted photocatalytic hydrogen production has recently attracted increasing attention [62]. In this strategy, the oxidation of organic substrates partially replaces the water oxidation reaction, alleviating kinetic limitations while coupling waste-resource valorization with hydrogen production [278,279,280]. For example, CdZnS-based systems incorporating single-atom cocatalysts can promote the photoreforming of organic substrates such as waste polymers and biomass derivatives, producing H_2_ together with value-added chemicals. This approach provides a new application direction for metal sulfide photocatalytic systems [60,79]. Overall, the development of HER systems has progressed from simply increasing the supply of photogenerated electrons toward the integrated optimization of electron transport, surface reaction kinetics, and system stability [48,55].

### 4.2. Photocatalytic CO_2_ Reduction

CO_2_ reduction uses solar energy to convert CO_2_ into value-added chemicals such as CO, formic acid, and methanol, providing an important pathway toward an artificial carbon cycle [281,282,283,284,285,286,287,288].

Compared with HER, CO_2_ reduction involves coupled multielectron and multiproton transfer processes and therefore depends more strongly on surface adsorption, electronic-structure regulation, and the stabilization of reaction intermediates [289,290,291,292,293,294]. Metal sulfides show considerable potential for CO_2_ adsorption, activation, and reduction because of their relatively negative conduction-band positions, strong visible-light response, and tunable surface electronic structures [105]. Among them, ZnIn_2_S_4_, CuInS_2_, and other Cu-based multinary sulfides have been extensively investigated for photocatalytic CO_2_ reduction owing to their diverse surface chemical environments and strong light-absorption capabilities [295,296,297].

Whereas HER primarily requires an efficient supply of photogenerated electrons, CO_2_ reduction is more sensitive to the ability of a catalyst to adsorb and activate CO_2_ and stabilize key reaction intermediates [95]. Regulation of surface defects, metal sites, and interfacial electronic structures can control the formation of key intermediates such as COOH*, CO*, and CHO*, thereby altering the selectivity toward the final products [97,105]. In defect-engineered ZnIn_2_S_4_, for example, sulfur vacancies can induce localized electron enrichment, strengthen CO_2_ adsorption, and promote the formation of COOH*, thereby accelerating CO_2_ reduction kinetics [49,97].

**Figure 10:**
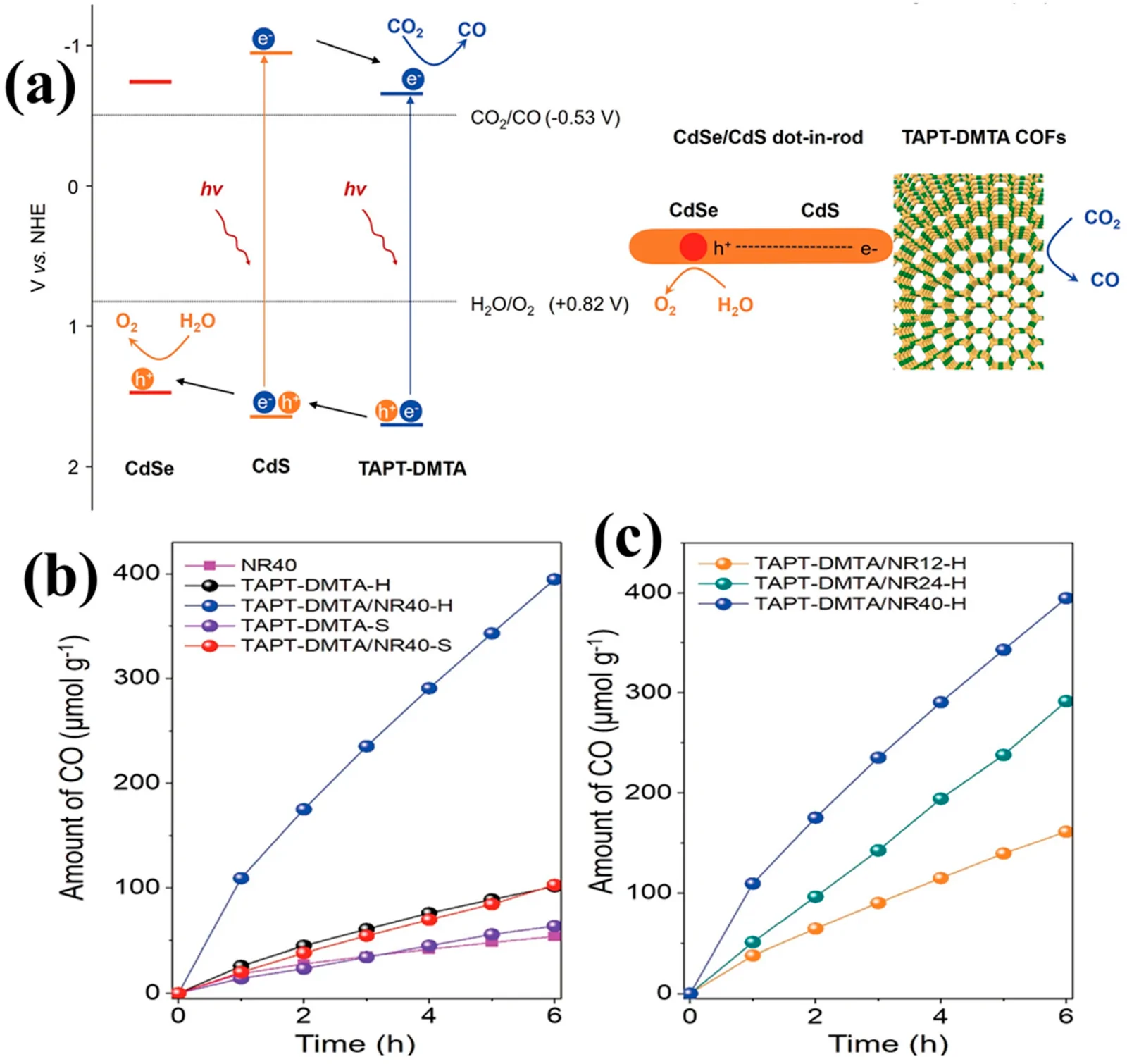
(**a**) Band structure diagrams of TAPT-DMTA, CdS, and CdSe, alongside the proposed CO_2_ photoreduction mechanism enabled by heterojunction structures in TAPT-DMTA/NR40-H nanocomposites, (**b**) Time-resolved CO_2_-to-CO conversion performances of NR40, TAPT-DMTA-H, TAPT-DMTA/NR40-H, TAPT-DMTA-S, and TAPT-DMTA/NR40-S catalysts, (**c**) Temporal CO_2_ photoreduction behaviors of TAPT-DMTA/NR12-H, TAPT-DMTA/NR24-H, and TAPT-DMTA/NR40-H composite samples, reproduced with permission from Ref. [298] Copyright 2022, Springer Nature.

S-scheme heterostructures such as ZnIn_2_S_4_/BiVO_4_ can further improve CO_2_ reduction performance by establishing an interfacial built-in electric field that drives the directional transfer of photogenerated electrons. This charge-transfer configuration improves charge-carrier separation while preserving a strong reduction capability [50,106]. Different metal sulfide systems exhibit distinct selectivities toward products such as CO, CH_4_, CH_2_OH, and HCOOH. These variations arise primarily from differences in surface electronic structure, CO_2_ adsorption strength, and the binding of key intermediates [105]. Cu-based sulfides, for instance, may offer advantages for multielectron transfer and the formation of multicarbon products because of their strong CO_2_-activation capability and favorable binding strengths for carbon-containing intermediates. By contrast, Zn- and In-based sulfides tend to favor the formation of single-carbon products such as CO and formic acid [97]. Such distinctions in band-edge positions, reaction pathways and intermediate-binding preferences across metal-sulfide photocatalysts are visualized in Figure 10.

Nevertheless, current photocatalytic CO_2_ reduction systems continue to face challenges associated with insufficient product selectivity, competition from side reactions, and difficulty in accurately closing the carbon balance [54]. Future research should integrate in situ diffuse reflectance infrared Fourier transform spectroscopy (DRIFTS), X-ray absorption spectroscopy (XAS), and theoretical calculations to identify operative reaction pathways and monitor the dynamic evolution of active sites. A more standardized framework for evaluating photocatalytic CO_2_ reduction performance is also required [54].

### 4.3. Photocatalytic H_2_O_2_ Production

Hydrogen peroxide (H_2_O_2_) is a green oxidant with important applications in environmental remediation, chemical synthesis, and energy storage [299,300,301,302]. Although the conventional anthraquinone process enables industrial-scale production, it involves relatively high energy consumption, operational complexity, and a substantial environmental burden. The direct synthesis of H_2_O_2_ through the solar-driven two-electron oxygen reduction reaction (2e^−^ ORR) has therefore attracted increasing attention [225,303].

Metal sulfides show considerable potential for photocatalytic H_2_O_2_ production because of their tunable electronic structures, strong visible-light response, and abundant surface-active sites. Materials such as ZnIn_2_S_4_, CdS, and SnS_2_ can promote O_2_ adsorption by regulating their conduction-band positions and surface electronic states. They can also lower the formation barrier of the OOH* intermediate, thereby improving the efficiency and selectivity of H_2_O_2_ production [49,225].

Unlike HER, H_2_O_2_ production depends strongly on the selective adsorption and activation of molecular oxygen; surface defect engineering is therefore an important strategy for controlling the reaction pathway. Sulfur vacancies can redistribute local electron density, enhance O_2_ adsorption on the catalyst surface, and facilitate the two-electron oxygen reduction pathway [49,225]. An excessive defect concentration, however, may cause overly strong adsorption of oxygen species, favor the four-electron reduction pathway, or promote other side reactions. Precise control over defect concentration and spatial distribution is therefore necessary to balance activity and selectivity [49,54]. In defect-engineered ZnIn_2_S_4_, for example, an appropriate concentration of sulfur vacancies enhances O_2_ activation while maintaining effective charge-carrier separation, resulting in high selectivity during photocatalytic H_2_O_2_ production [225]. Heterostructure construction can further regulate interfacial electron transfer and direct photogenerated electrons preferentially toward oxygen reduction, thereby increasing the H_2_O_2_ production rate [50].

The desired 2e^−^ ORR competes with the 4e^−^ route to water (or hydroxide), which can be thermodynamically favorable but consumes the O–O bond and lowers H_2_O_2_ selectivity. The adsorption geometry of O_2_ is one factor: end-on adsorption often favors retention of the O–O bond and formation/desorption of *OOH, whereas side-on adsorption can facilitate O–O cleavage and deeper reduction. The d-band center of a metal active site can influence O_2_ and *OOH binding, but its effect is not universal and must be evaluated with coordination, oxidation state, and the sulfide support. A useful design should stabilize *OOH sufficiently for proton-coupled electron transfer while avoiding excessively strong binding that promotes O–O cleavage or H_2_O_2_ decomposition. Faradaic/selectivity measurements, H_2_O_2_ decomposition controls, and operando evidence should accompany DFT assignments.

**Figure 11:**
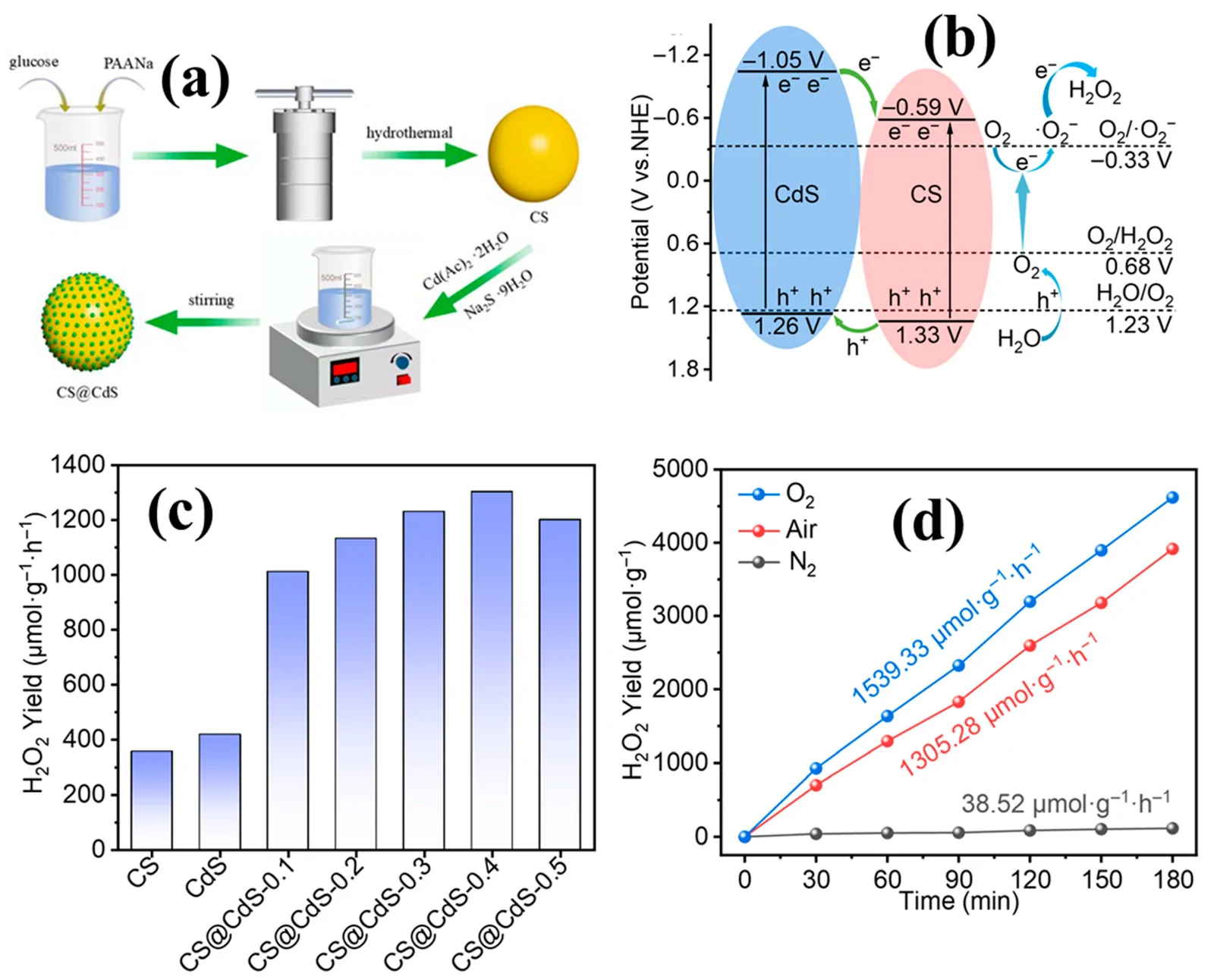
(**a**) Schematic illustration of the synthetic route for CS@CdS composites, (**b**) Photocatalytic H_2_O_2_ generation mechanism of the CS@CdS photocatalyst, (**c**) Visible-light-driven H_2_O_2_ production performance of CS@CdS, (**d**) Kinetic curves of photocatalytic H_2_O_2_ yields over CS@CdS under nitrogen, oxygen, and ambient air conditions, reproduced with permission from Ref. [304], MDPI.

In the work of He et al., a novel heterojunction photocatalyst constructed with cadmium sulfide nanoparticles anchored on carbon spheres (CS@CdS) was successfully designed and fabricated (Figure 11). The prepared CS@CdS composite delivers a prominent photocatalytic H_2_O_2_ generation rate of 1305 μmol·g^−1^·h^−1^ in pure water under ambient air conditions, without the assistance of sacrificial reagents or external oxygen supplementation. This optimized catalytic activity is 3.1-fold and 3.6-fold higher than those of pristine CdS and bare carbon spheres (CS), respectively. Comprehensive structural and photoelectrochemical characterizations demonstrate that the intimate interfacial coupling between CS and CdS establishes a valid electronic junction. Such a well-designed heterostructure greatly accelerates the spatial separation of photogenerated charge carriers and extends the carrier lifespan, which collectively contributes to the remarkably enhanced photocatalytic H_2_O_2_ production efficiency [304].

In recent years, integration with carbon materials and the design of porous architectures have also been employed to improve H_2_O_2_ production over metal sulfides [77,81]. Carbon-based supports can enhance catalyst dispersion and interfacial electron transport while facilitating catalyst recovery, offering new possibilities for future immobilized systems [305]. Nevertheless, most studies of photocatalytic H_2_O_2_ synthesis remain focused on performance evaluation under laboratory conditions. Long-term continuous operation, product separation, and the design of scalable reactors remain important challenges for future development [77,277].

### 4.4. Environmental Remediation

Industrial production, traffic transportation, agricultural production and urban domestic activities discharge a variety of pollutants, including persistent organic substances, toxic metal ions, pathogenic microorganisms, nitrogen oxides, sulfur-containing gaseous pollutants and volatile organic compounds [168,306,307,308,309,310,311,312,313,314,315,316,317]. Traditional pollutant treatment technologies often merely shift contaminants across different physical phases or fail to eliminate trace-level pollutants thoroughly [318,319,320,321,322,323,324,325,326,327,328]. As an efficient photocatalytic technology, metal sulfides can produce electrons, holes and reactive oxygen species upon visible light irradiation, which enables pollutant reduction, oxidation degradation, mineralization and microbial sterilization effects. Nevertheless, multiple practical limiting factors need to be considered in real-world applications, such as mass transfer efficiency, environmental humidity, natural organic matter interference, coexisting ion effects, catalyst recycling performance, the toxicity of pollutant transformation byproducts, and overall energy consumption [329,330,331]. In these applications, photogenerated electrons and holes produce reactive species such as superoxide radicals (•O_2_^−^) and hydroxyl radicals (•OH), enabling the degradation of organic contaminants, the reductive transformation of heavy metals, and the treatment of complex contaminated matrices [332,333,334].

Early studies focused primarily on dye degradation. Sulfides such as CdS, ZnS, and ZnIn_2_S_4_ can promote the formation of reactive oxygen species through photogenerated charge carriers, resulting in the rapid degradation of model contaminants such as methylene blue and rhodamine B [46]. As the field has developed, however, research targets have shifted from simple dye systems toward antibiotics, pesticides, organophosphorus contaminants, and real wastewater matrices. These more complex applications impose greater demands on catalyst selectivity, stability, and resistance to matrix interference [335,336,337,338].

For antibiotic remediation, photogenerated redox-active species produced by metal sulfides can attack aromatic structures and key functional groups, leading to the stepwise oxidation and eventual mineralization of the contaminants [104]. Systems based on ZnIn_2_S_4_, Bi_2_S_3_, and SnS_2_, for example, have been applied to the degradation of tetracycline and sulfonamide antibiotics. Defect engineering and heterostructure construction can improve charge-carrier separation and promote the generation of reactive species in these systems [335,336,339].

In recent years, the remediation of agricultural pollution has emerged as an important extension of environmental photocatalysis. Livestock and aquaculture wastewater, pesticide residues, and irrigation return flows generated during agricultural production commonly contain complex mixtures of organic contaminants, nitrogen- and phosphorus-containing nutrients, inorganic ions, and natural organic matter. Simple model-contaminant systems therefore cannot adequately represent practical treatment conditions [64,127,248]. Compared with single-contaminant systems, coexisting constituents in complex agricultural water matrices may compete for adsorption at active sites and interfere with charge-carrier transport and reactive-species generation. Greater attention must therefore be devoted to catalyst resistance to matrix interference and long-term operational stability [238,248].

The main matrix effects are competitive and concentration-dependent. Bicarbonate can intercept •OH and convert it to the less reactive but more selective CO_3_•^−^; chloride can scavenge •OH and, under some conditions, form reactive chlorine species with different selectivity and possible by-products. Humic substances absorb light, quench radicals, and compete for surface sites, although their effect can vary with molecular composition and concentration. Biochar supports may partly mitigate this competition by enriching hydrophobic or aromatic target contaminants near the photocatalyst, but adsorption alone does not establish degradation, and strong binding can impede regeneration. Matrix-specific adsorption, transformation products, toxicity, and carbon balances should therefore be evaluated in real wastewater.

To address the requirements of complex water treatment, metal sulfides can be integrated with carbon materials, porous supports, or membrane materials to enhance pollutant enrichment and facilitate catalyst separation and recovery [340,341,342]. Agricultural-waste-derived carbon materials are particularly promising as sustainable photocatalyst supports because of their wide availability, relatively low cost, and structural tunability [341,343,344]. Agricultural residues such as straw, nutshells, and other by-products can be converted through pyrolysis and activation into biochar materials with well-developed porous structures and abundant surface functional groups. Modified biochar itself can achieve efficient enrichment of multiple antibiotic pollutants from wastewater relying on abundant surface sites [345]. These materials enhance pollutant adsorption while promoting the dispersion and immobilization of the semiconductor photocatalysts [343,346]. Biomass-derived carbon materials can also improve interfacial electron transport, thereby increasing charge-carrier utilization in metal sulfide photocatalytic systems [347].

In addition to organic-contaminant degradation, heavy-metal remediation is an important application of metal sulfides [348]. Because sulfide semiconductors generally possess relatively negative conduction-band positions, their photogenerated electrons can drive the reductive transformation of highly toxic metal species such as Cr(VI) and Hg(II), thereby reducing toxicity and potentially facilitating resource recovery. Environmental remediation systems nevertheless continue to face challenges arising from low pollutant concentrations, interference by coexisting ions, and inadequate long-term catalyst stability [67]. Beyond organic and heavy-metal abatement, pathogenic biofilm contamination on food-contact surfaces also poses serious public-health risks, for which combined essential-oil and cold-plasma disinfection strategies have been reported [349,350]. Future research should therefore incorporate evaluations using real water samples, catalyst-immobilization technologies, and continuous-flow reaction systems to improve the applicability of metal sulfide photocatalysis under complex environmental conditions [67,238].

### 4.5. Real-Water Treatment, Catalyst Immobilization, and Practical Applications

Although powdered metal sulfide photocatalysts exhibit high activity under laboratory conditions, their practical application remains constrained by difficulties in catalyst recovery, inadequate long-term stability, and the risk of secondary contamination [27,351,352]. Immobilizing powdered catalysts on stable supports to combine efficient photocatalytic reactions with convenient catalyst separation is therefore an important step toward the engineering implementation of photocatalytic technologies [353].

**Figure 12:**
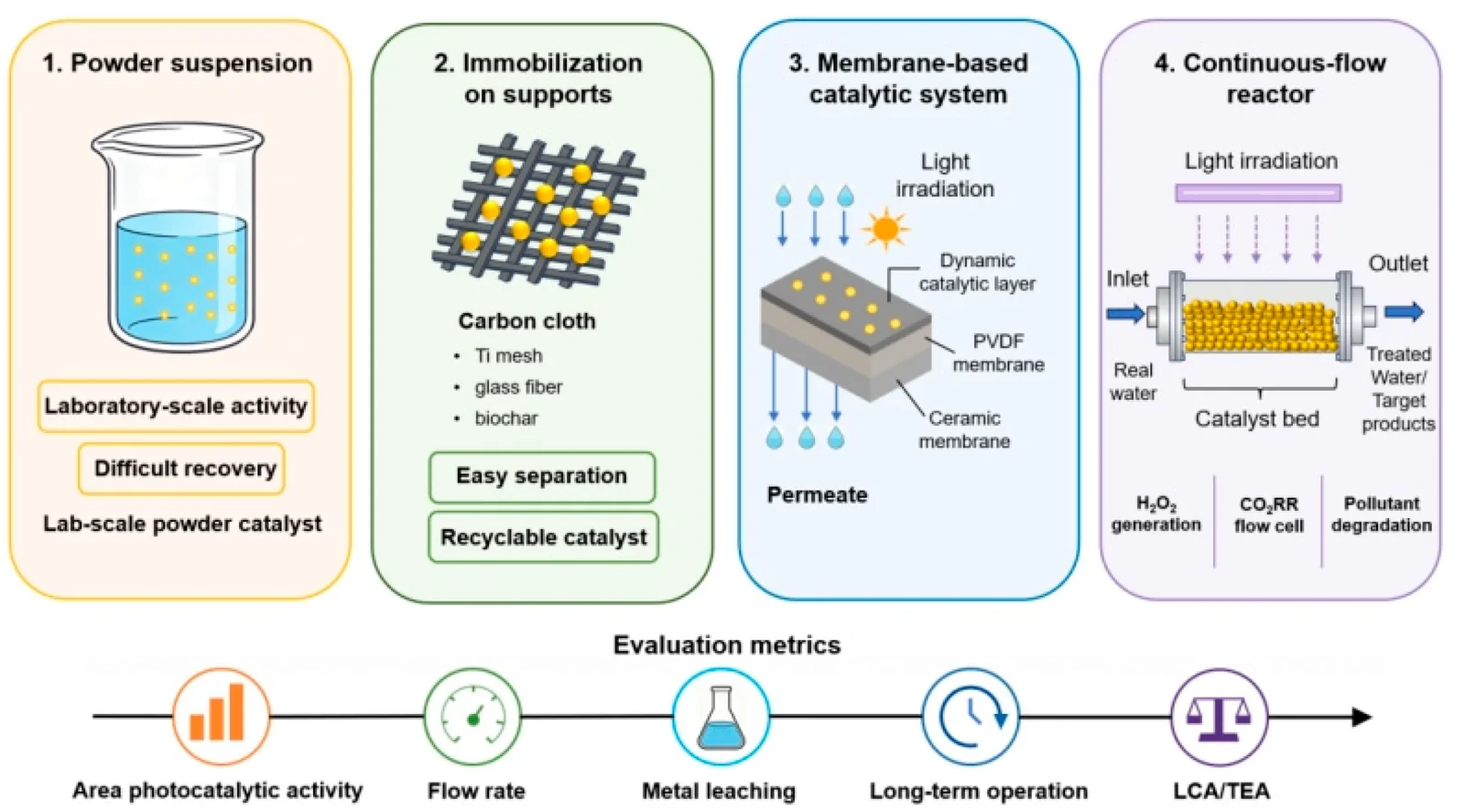
Immobilization routes from powdered sulfides to membranes, meshes, carbon supports, and continuous-flow reactors.

The progressive transition from lab-scale powder catalysts toward practically viable reaction systems is visually summarized in Figure 12. It outlines four representative configurations: powder suspension, support-immobilized catalysts, membrane-based catalytic layers, and continuous-flow reactors, alongside key evaluation metrics for assessing real-world performance.

Common immobilization approaches include loading catalysts onto carbon materials, anchoring them to metal meshes, incorporating them into membrane architectures, and coupling them with porous supports [353,354]. Among these approaches, carbon-based materials have been extensively investigated for the immobilization of metal sulfides because of their favorable electrical conductivity, chemical stability, and diverse surface structures [340].

Agricultural-waste-derived carbon materials offer potential advantages as sustainable supports owing to their wide availability, low cost, and structural tunability [341,343]. Agricultural residues such as rice straw and corn straw can be converted by pyrolysis into porous carbon structures. These materials provide a promising route for photocatalyst immobilization by enhancing pollutant enrichment, improving catalyst dispersion, and increasing cycling stability [343,346]. Catalyst recovery can be further facilitated by incorporating magnetic components. For example, agricultural-waste-derived magnetic carbon materials containing Fe_2_O_4_ or related magnetic components can couple pollutant adsorption with magnetic separation, thereby improving material recovery and reuse during the treatment of complex aqueous matrices [347,355,356].

It should be emphasized that agricultural-derived materials primarily serve as low-cost supports and functional auxiliary components in metal sulfide photocatalytic systems rather than as replacements for the catalytically active metal sulfides [355]. Future practical applications must continue to address the stability of active sulfide components, the risk of metal-ion leaching, and the design of continuous-flow reactors [27,353]. In real matrices such as livestock and aquaculture wastewater, agricultural runoff, and rural domestic sewage, long-term operational testing and pilot- or larger-scale validation will be required to facilitate the transition of metal sulfide photocatalysis from laboratory research to practical environmental remediation [238,248,357].

### 4.6. Photoelectrochemical Monitoring and Sensing Applications

With the rapid advancement of modern industrial systems, there has been a growing demand for high-performance gas sensing devices applied in environmental surveillance and industrial safety supervision [358,359,360,361,362,363,364,365,366,367,368]. Despite such technological development, conventional chemical gas sensors still suffer from inherent drawbacks including high energy consumption and insufficient detection sensitivity, which are primarily attributed to the poor charge transfer efficiency of traditional gas-sensitive functional materials [369,370,371]. As a multifunctional semiconductor material, metal sulfides can function simultaneously as a visible-light harvester and a chemically modifiable signal transducer. Featuring low fabrication cost, visible-light responsiveness, nitrogen-enriched surface-active sites and excellent heterojunction construction compatibility, this material has emerged as a promising candidate for low-power gas sensors and photoelectrochemical biosensors [372]. Notably, the superior physicochemical properties that support its excellent photocatalytic activity, including prominent light absorption capacity, prolonged photogenerated carrier lifespan, rapid interfacial charge migration and specific surface adsorption characteristics, are also conducive to boosting the sensing accuracy and lowering the limit of detection of relevant sensor devices [373,374,375,376,377,378,379,380,381,382].

**Figure 13:**
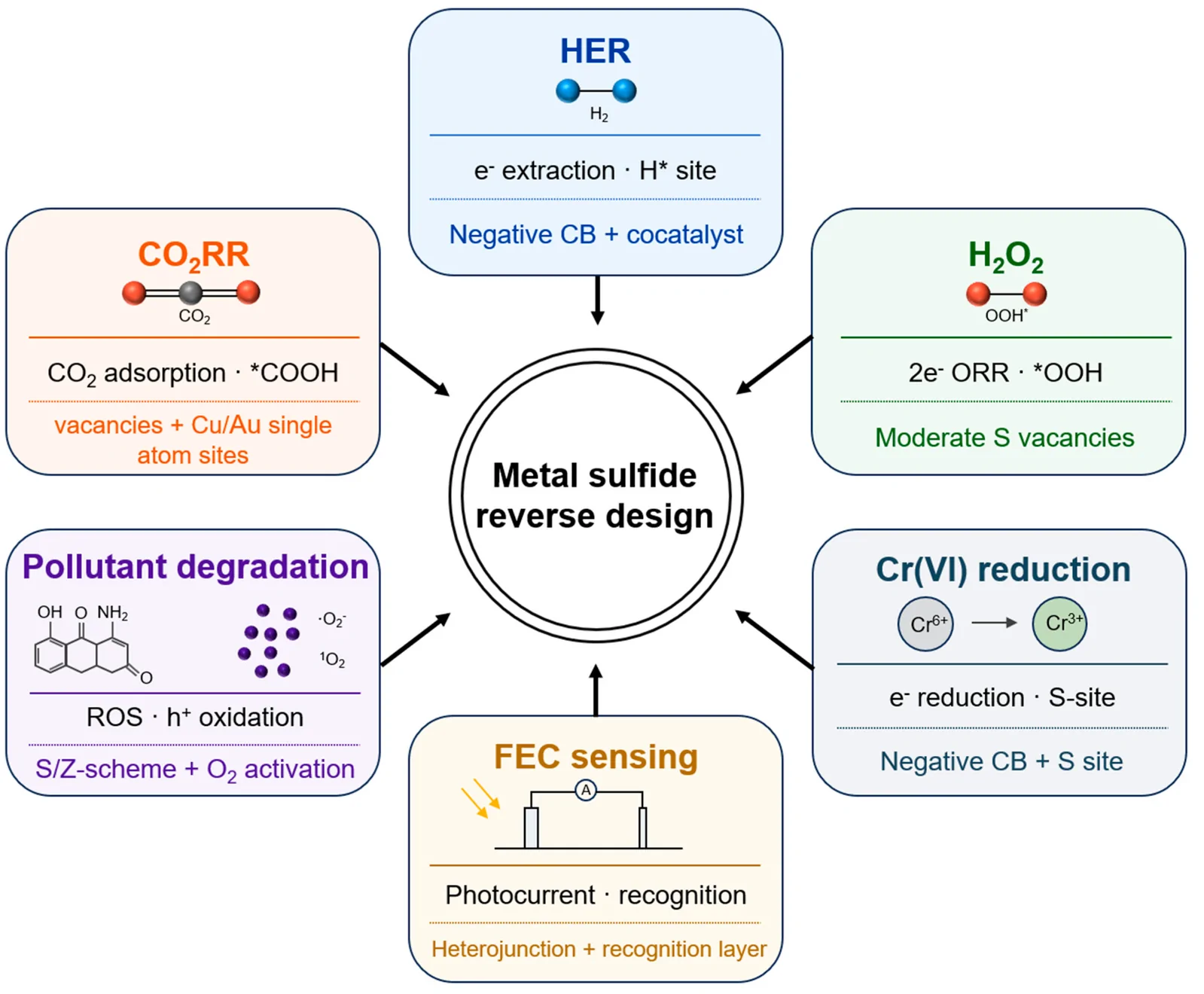
Correspondence among charge requirements, reaction sites, and structural regulation strategies in different applications.

Beyond energy conversion and environmental remediation, the favorable photoelectric conversion properties of metal sulfides have made them an important class of materials for photoelectrochemical (PEC) sensing [383,384,385,386]. Compared with conventional analytical methods, PEC sensing couple’s photoexcitation with electrochemical signal transduction and offers advantages such as high sensitivity, rapid response, and relatively simple instrumentation [387,388,389,390,391,392]. Such structure-function correlations across diverse reaction scenarios are generalized in Figure 13, which maps target applications, intrinsic charge demands, active-site requirements and corresponding structural modulation strategies for metal sulfide reverse design.

Sulfide materials such as CdS, ZnIn_2_S_4_, and Bi_2_S_3_ have been extensively investigated for detecting contaminants, ions, and biomolecules because of their strong light absorption, tunable electronic structures, and favorable photoelectrochemical responses [78,393]. CdS, with its efficient light absorption and electron transport, was a representative sulfide semiconductor in early PEC sensing studies. Cd-free sulfides such as ZnIn_2_S_4_ and Bi_2_S_3_, by contrast, have increasingly been explored for environmental contaminant detection because of their relatively high stability and structural tunability [393].

To enhance PEC sensing performance, heterostructure construction, defect regulation, and surface functionalization are commonly employed to improve photogenerated charge-carrier separation and target-recognition capability [393]. Sulfide/oxide heterostructures, for example, can enhance the photocurrent response through interfacial charge transfer. Defects such as sulfur vacancies can increase the number of surface-active sites, strengthen the adsorption of contaminant molecules, and improve detection sensitivity [28,393].

In recent years, metal sulfide-based PEC sensing has gradually progressed from measurements in laboratory model systems toward the analysis of complex environmental samples. The integration of functional recognition layers, nanocomposite architectures, and miniaturized sensing platforms can enable rapid detection of heavy-metal ions, organic contaminants, and biological targets, providing new technical approaches to environmental monitoring [348,393]. Nevertheless, current research on metal sulfide PEC sensors remains focused primarily on sensitivity evaluation under laboratory conditions. Their long-term stability, resistance to matrix interference, and suitability for on-site analysis in complex environmental samples require further investigation [54,394]. Future development should integrate stability-oriented structural design with testing in real environmental samples and portable sensing platforms, facilitating the transition from highly sensitive laboratory systems to practical environmental monitoring applications [77]. Magnetic nanoparticles have exhibited prominent advantages as multifunctional supports for immunosensors and aptasensors toward mycotoxin analysis in complex food matrices [395], which inspires the integration of magnetic separation units into metal sulfide-based PEC sensing platforms. Aptamer-based upconversion sensors can also realize highly sensitive determination of tetracycline residues in real food samples [396]. Furthermore, the aptamer-sandwich recognition strategy, which has been proven effective for pathogenic bacteria quantification in practical food samples, offers valuable inspiration for broadening the analyte range of metal sulfide-based PEC biosensors [397]. SERS-aptasensor platforms have also demonstrated feasibility for rapid screening of Salmonella typhimurium in food matrices [398].

## 5. Challenges and Future Perspectives

Despite substantial progress in metal sulfide photocatalysts for energy conversion and environmental remediation, their further development remains hindered by an incomplete understanding of reaction mechanisms, limited long-term stability, a lack of standardized evaluation protocols, and barriers to engineering implementation. Future research should integrate material structure design, analysis of dynamic reaction processes, and practical application requirements to establish a comprehensive research framework spanning fundamental mechanisms and engineering applications [27,54,277].

### 5.1. Mechanistic Understanding and Standardized Evaluation

**Figure 14:**
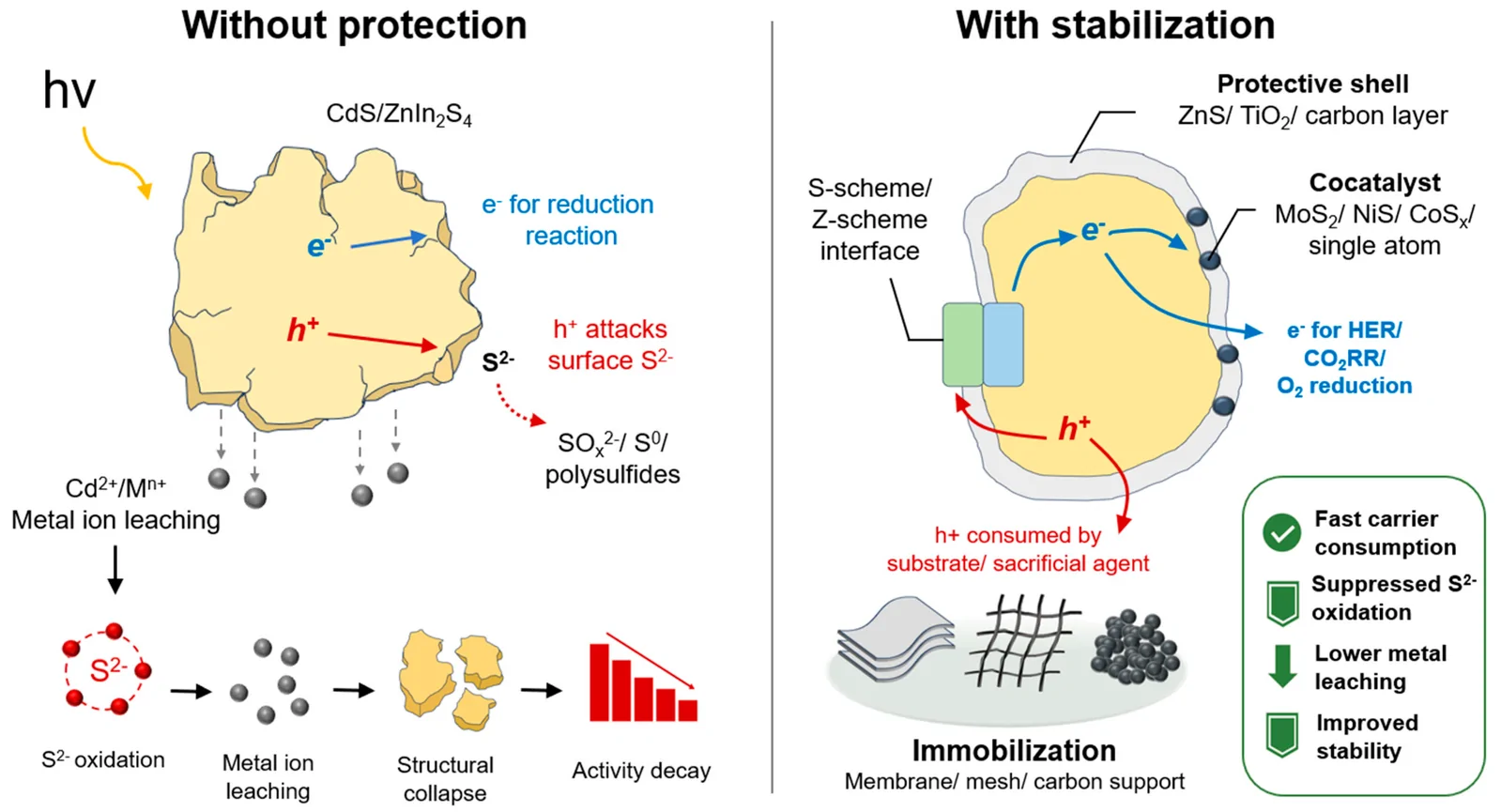
Photocorrosion pathways and structural stabilization strategies of metal sulfide photocatalysts.

In recent years, defect engineering, heterostructure construction, and cocatalyst modification have substantially improved the photocatalytic performance of metal sulfides [28,58,340]. Nevertheless, a unified understanding of the intrinsic relationships between structural regulation and catalytic performance has yet to be established [399,400,401]. In complex interfacial systems, in particular, the transport pathways of photogenerated charge carriers, the identity of surface reaction sites, and the processes of dynamic structural evolution remain under debate [27,402] as shown in Figure 14. Advanced characterization techniques, including in situ X-ray photoelectron spectroscopy (XPS), time-resolved spectroscopy, surface photovoltage measurements, and in situ absorption spectroscopy, have been extensively employed to investigate charge generation, transport, and reaction processes in metal sulfides. These techniques provide an important basis for establishing structure–performance relationships [403,404,405]. In situ XPS, for example, can reveal changes in elemental oxidation states and interfacial electron transfer under irradiation. X-ray absorption spectroscopy (XAS) and in situ infrared spectroscopy can further probe the evolution of active sites and the formation of reaction intermediates, providing direct evidence for identifying operative catalytic pathways [405,406]. Metal sulfide surfaces, however, commonly contain defects, interfacial regions, and multiple types of exposed active sites simultaneously. It therefore remains difficult to distinguish the individual contributions of these structural features to catalytic performance [27]. Future studies should integrate multiscale in situ characterization, theoretical calculations, and data-driven analytical methods to shift the emphasis from static structural characterization toward the analysis of dynamic reaction processes [403,404].

Substantial differences also remain in the evaluation of photocatalytic performance across different studies [58]. Reported hydrogen evolution rates, CO_2_ reduction yields, and pollutant degradation efficiencies are strongly influenced by factors such as light-source characteristics, catalyst concentration, reaction conditions, and measurement duration. Direct comparison among studies is therefore often difficult. Establishing more standardized evaluation criteria—including apparent quantum efficiency (AQE), solar-energy conversion efficiency, product selectivity, long-term cycling stability, and elemental mass-balance analysis—is essential for advancing research on metal sulfide photocatalysis [54].

In environmental remediation, systems containing a single model contaminant cannot adequately represent the complexity of real aqueous environments. Actual wastewater commonly contains multiple organic contaminants, inorganic ions, and natural organic matter, all of which may affect pollutant adsorption, interfacial reactions, and photogenerated charge-carrier transport [54]. Agricultural water matrices present additional challenges because livestock and aquaculture wastewater, agricultural runoff, and irrigation return flows commonly exhibit complex contaminant compositions, pronounced seasonal variation, and strong ionic interference. Long-term evaluation under conditions more representative of practical applications is therefore required [238,248]. Future studies should strengthen performance assessment in real water matrices, including long-term operational testing in agricultural, industrial, and domestic wastewater. Immobilized photocatalytic systems and continuous-flow devices should also be employed to validate the practical potential of these materials [77,357].

### 5.2. Photocorrosion, Safety, and Environmental Compatibility

Insufficient stability remains one of the principal barriers to the practical application of metal sulfide photocatalysts [58,67,407]. Compared with oxide semiconductors, some metal sulfides offer stronger visible-light absorption and greater electron reduction capability. However, lattice S^2−^ species are susceptible to oxidation by photogenerated holes, resulting in sulfur loss, structural degradation, and declining catalytic activity, as presented in Figure 14. In representative sulfide systems such as CdS, photocorrosion not only causes a progressive loss of activity but may also release Cd^2+^ into the surrounding medium, raising potential environmental safety concerns [58,67]. Major photodegradation pathways for metal sulfides and available stabilization strategies are summarized in Figure 14. The diagram contrasts uncontrolled photocorrosion that causes metal-ion leaching and structural collapse with multiple mitigation approaches such as protective shell coating, heterojunction construction, cocatalyst loading and substrate immobilization.

Coating metal sulfides with protective layers, constructing heterostructures, and introducing cocatalysts are therefore among the principal strategies used to suppress photocorrosion and improve material stability [67,340,402]. In a CdS/ZnS core–shell structure, for example, the stable ZnS shell can mitigate surface oxidation of CdS while maintaining interfacial electron transfer, thereby improving long-term photocatalytic stability [408].

Heterointerfaces and cocatalysts can additionally facilitate directional charge transfer and limit the accumulation of photogenerated holes at sulfide surfaces, thus slowing photocorrosion [340,402]. Most stability assessments, however, remain limited to short-term cycling experiments. Systematic investigations of structural changes, defect evolution, and metal-ion release under prolonged irradiation remain scarce [27]. Future studies should therefore integrate in situ characterization with long-term operational testing to elucidate the dynamic evolution of metal sulfides under realistic reaction conditions [403,404].

Beyond conventional stabilization strategies, emerging composition designs offer further possibilities for improving the durability of metal sulfides. High-entropy sulfides, for instance, exploit interactions among multiple metal elements to regulate local lattice environments and electronic structures. They may therefore improve catalytic activity and structural stability simultaneously, providing a potential design route toward high-performance, long-lived photocatalytic materials [409,410]. Improvements in catalyst stability should not, however, be evaluated solely in terms of performance retention; material cost, environmental impacts, and recyclability must also be considered [411]. Life-cycle assessment (LCA) and techno-economic analysis (TEA) can provide a broader evaluation of metal sulfide systems across material production, operation, and end-of-life treatment, thereby informing the development of more sustainable catalytic materials.

### 5.3. Transition from Laboratory Systems to Practical Applications

Most current studies of metal sulfide photocatalysis remain based on laboratory-scale slurry systems containing suspended catalyst powders. Their practical implementation is constrained by difficulties in catalyst recovery, performance deterioration during long-term operation, and limited compatibility with engineered reactors [353]. Developing efficient immobilization strategies and appropriately designed reactor systems is therefore critical to the continued advancement of metal sulfide photocatalysis [412,413]. Compared with suspended powders, immobilized systems can reduce catalyst-separation costs, improve catalyst recovery and reuse, and offer greater suitability for continuous-flow operation. Common immobilization approaches include loading catalysts onto carbon materials, anchoring them to metal meshes, incorporating them into porous membranes, and constructing three-dimensional supports. Carbon-based materials have attracted particular attention as supports for immobilized metal sulfide photocatalysts because of their favorable electrical conductivity, chemical stability, and structural tunability [340].

Agricultural-waste-derived carbon materials have recently shown promise as sustainable supports because of their wide availability, relatively low cost, and tunable pore structures [341,343]. Porous carbon materials prepared from agricultural by-products such as straw and nutshells can enhance pollutant enrichment, improve catalyst dispersion, and increase material recovery and reusability, providing an alternative design strategy for metal sulfide immobilization [346]. Magnetic biochar composite supports can also be constructed by incorporating magnetic components, thereby coupling photocatalytic reactions with material recovery. For example, agricultural-waste-derived magnetic carbon materials containing components such as Fe_2_O_4_ can improve separation efficiency during the treatment of complex aqueous matrices [347,355].

It should be recognized, however, that agricultural-derived materials primarily function as low-cost supports and auxiliary functional components in metal sulfide photocatalytic systems [414,415,416]. Their main purpose is to improve catalyst dispersion, stability, and recoverability at the engineering scale rather than to replace the catalytically active metal sulfide components. Future metal sulfide photocatalytic systems should be integrated with fixed-bed reactors, membrane reactor systems, and continuous-flow devices to facilitate the transition from laboratory-scale powdered catalysts to engineered treatment systems [353,411]. Evaluation protocols should also account for long-term operation in real water samples, the coexistence of complex contaminant mixtures, and seasonal variations in water quality [238,248,353].

### 5.4. Sustainable Design and Future Development Directions

As metal sulfide systems become increasingly complex, conventional experience-driven approaches to material development are becoming less capable of meeting the demand for rapid screening of efficient photocatalysts [417]. Machine learning, high-throughput computation, and database-driven design have recently been introduced into photocatalytic materials research to enable material screening and performance prediction by establishing relationships among structural parameters, electronic properties, and catalytic performance. For example, machine-learning analysis of key descriptors—including material composition, defect structures, band parameters, and adsorption energies—can assist in identifying new sulfide systems with potential practical value while reducing the cost of experimental exploration.

The development of reliable artificial intelligence models, however, depends on access to high-quality experimental databases [418,419,420]. Inconsistent testing conditions and nonuniform data formats remain prevalent in photocatalysis research, limiting model accuracy and transferability. Establishing standardized photocatalysis databases would facilitate the integration of experimental data, theoretical calculations, and engineering assessment information, thereby providing a robust data foundation for intelligent materials design. Furthermore, combining artificial intelligence methods with high-throughput experimentation and first-principles calculations may accelerate the transition of metal sulfide design from empirical exploration toward data-informed prediction [421].

Future catalyst evaluation should also extend beyond laboratory-scale activity to consider resource consumption, environmental impacts, and the cost of large-scale production [411]. The integration of life-cycle assessment (LCA) and techno-economic analysis (TEA) can enable a more comprehensive evaluation of metal sulfide photocatalytic systems across material production, operation, recovery, and reuse. Such analyses can provide broader guidance for the future development of sustainable catalytic materials.

## 6. Conclusions

Metal sulfide photocatalysts offer broad compositional and structural flexibility, but their performance is determined by the match between electronic structure, active-site chemistry, charge-carrier kinetics, and the target reaction. Morphology, defects, doping, interfaces, and cocatalysts can each improve a particular step; none guarantees an overall gain if the modification also accelerates recombination, weakens selectivity, or destabilizes the sulfide lattice. The evidence reviewed here supports reaction-specific design and mechanistic verification under operating conditions rather than relying on ex situ structure or a single activity metric.

Several issues remain central to practical development. Photocorrosion must be assessed through long-term activity, structural evolution, and dissolved-metal analysis, especially for Cd-containing materials. Donor-assisted HER should be distinguished from overall water splitting, and performance reports should include comparable illumination, product-specific rates, selectivity, AQE where possible, and mass balance. For environmental treatment, real-water tests must account for radical scavenging and competitive adsorption by inorganic ions and natural organic matter. Immobilized sulfides and agricultural-waste-derived carbon supports may improve recovery and target enrichment, but their contribution should be validated in continuous operation and not inferred from adsorption alone. A useful next step is to combine operando characterization, isotope and product controls, realistic matrix testing, and consistent performance reporting. This will help determine which structural features persist during reaction and whether laboratory improvements translate into selective, stable, and environmentally acceptable photocatalytic systems.

## Figures and Tables

**Table 1:** Typical metal sulfides and their intrinsic properties.

Material	Band Gap	Structure	Advantage	Limitation	Suitable Reaction
CdS	~2.4 eV, relatively negative conduction band	Binary sulfide; commonly synthesized as nanorods or nanoparticles	Strong visible-light response; high activity toward HER	Photocorrosion; cadmium toxicity; risk of hole-induced oxidation	HER; heterojunction construction
ZnIn_2_S_4_	~2.4 eV, relatively negative conduction band	Layered ternary sulfide	Tunable morphology and defects; cadmium-free	Rapid charge recombination; susceptibility to photocorrosion	HER, CO_2_ RR, H_2_O_2_ production, pollutant degradation
Zn_x_Cd_1−x_S	Continuously tunable band gap and band edges	CdS–ZnS solid solution	Balanced light harvesting and reduction capacity	Cadmium hazard; insufficient phase stability; particle aggregation	HER, CO_2_RR
In_2_S_3_	~2.0–2.1 eV	Visible-light-responsive ternary sulfide	Relatively low toxicity	Limited electron–hole separation efficiency; facile charge recombination	Pollutant degradation, heavy metal reduction, heterojunction fabrication
SnS_2_	~2.2 eV, layered structure	Cadmium-free sulfide	Satisfactory stability; abundant layered active sites	Intrinsically low catalytic activity	Pollutant degradation, CO_2_RR, composite heterostructures
CuInS_2_	Narrow band gap with accessible Cu active sites	Ternary Cu-based sulfide	Great potential for modulating CO_2_ reaction intermediates	Complicated selectivity behavior; surface reconstruction	CO_2_RR, heterojunction construction
SnS_2_	~2.2–2.4 eV	Layered binary sulfide	Visible-light response; exposed layered surfaces	Carrier recombination; condition-dependent surface oxidation	CO_2_ reduction; H_2_O_2_; pollutant conversion
MoS_2_	~1.2 eV (bulk); larger in monolayer	Layered binary sulfide	Edge sites can promote proton reduction; thickness-tunable gap	Limited basal-plane activity; phase and edge dependence	HER; cocatalyst or heterojunction component
WS_2_	~1.3–2.0 eV (phase/thickness dependent)	Layered binary sulfide	Visible-light absorption and tunable edge structure	Phase-dependent transport and limited stability in some systems	HER; heterojunction construction
CuInS_2_	~1.5 eV	Ternary chalcopyrite sulfide	Strong visible-light absorption; adjustable composition	Surface oxidation and charge recombination	CO_2_ reduction; H_2_ evolution

**Table 2:** Comparison of structural regulation strategies.

Strategy	Main Function	Typical Example	Applicable Applications	Limitation
Morphology	Shorten charge carrier migration distance, expose more active sites and mass transport channels	CdS nanorods, ZnIn_2_S_4_ nanosheets, hollow/flower-like structures	HER, degradation, PEC monitoring	Accelerated corrosion under high specific surface area; difficult scale-up via template methods
Defect	Regulate local charge density, adsorption energy and intermediate stability	S-vacancy ZnIn_2_S_4_, dual defects, TiO_2_@ZnIn_2_S_4_	CO_2_RR, H_2_O_2_ production, pollutant degradation	Excessive defects generate recombination centers and impair stability
Composition	Tune bandgap, band edge positions and multi-metal active sites	ZnCdS solid solution, CuInS_2_, high-entropy sulfides	HER, CO_2_RR	Impurity phase formation, element segregation and toxicity risks
Interface	Construct built-in electric field to facilitate directional charge transfer	S/Z-scheme heterojunction, Schottky junction, p-n junction	HER, CO_2_RR, degradation, H_2_O_2_ production	Ambiguous charge transfer pathways; operando characterizations required for verification
Cocatalyst	Lower surface reaction energy barriers, optimize adsorption of H* or CO_2_ intermediates	MoS_2_, NiS, Pd single atoms, Au_1−x_S	HER, CO_2_RR	Light shielding, nanoparticle aggregation and active site migration

**Table 3:** Representative applications of metal sulfides.

Reaction	Material	Strategy	Performance Indicator	Challenge
HER	CdS/NiCoB	cocatalyst	H_2_ evolution	stability
CO_2_RR	ZnIn_2_S_4_/BiVO_4_	S-vacancy + S-scheme	selectivity	carbon balance
H_2_O_2_	S-vacancy-ZnIn_2_S_4_	defect	H_2_O_2_ yield	decomposition
Wastewater	In_2_S_3_/MOF	heterostructure	removal efficiency	real water

## Data Availability

All the data cited in this paper are derived from published literature and no new experimental data were generated. This original diagram was prepared with the assistance of Zhenjiang Weili Technology Co., Ltd.
